# Transcription profiling of lung adenocarcinomas of c-myc-transgenic mice: Identification of the c-myc regulatory gene network

**DOI:** 10.1186/1752-0509-2-46

**Published:** 2008-05-22

**Authors:** Susanne Reymann, Jürgen Borlak

**Affiliations:** 1 Fraunhofer Institute of Toxicology and Experimental Medicine (Fh-ITEM), Center for Drug Research and Medical Biotechnology, Nikolai-Fuchs-Str. 1, 30625 Hannover, Germany; 2 Center of Pharmacology and Toxicology, Hannover Medical School, Carl-Neuberg-Str. 1, 30625 Hannover, Germany

## Abstract

**Background:**

The transcriptional regulator c-Myc is the most frequently deregulated oncogene in human tumors. Targeted overexpression of this gene in mice results in distinct types of lung adenocarcinomas. By using microarray technology, alterations in the expression of genes were captured based on a female transgenic mouse model in which, indeed, c-Myc overexpression in alveolar epithelium results in the development of bronchiolo-alveolar carcinoma (BAC) and papillary adenocarcinoma (PLAC). In this study, we analyzed exclusively the promoters of induced genes by different in silico methods in order to elucidate the c-Myc transcriptional regulatory network.

**Results:**

We analyzed the promoters of 361 transcriptionally induced genes with respect to c-Myc binding sites and found 110 putative binding sites in 94 promoters. Furthermore, we analyzed the flanking sequences (+/- 100 bp) around the 110 c-Myc binding sites and found Ap2, Zf5, Zic3, and E2f binding sites to be overrepresented in these regions. Then, we analyzed the promoters of 361 induced genes with respect to binding sites of other transcription factors (TFs) which were upregulated by c-Myc overexpression. We identified at least one binding site of at least one of these TFs in 220 promoters, thus elucidating a potential transcription factor network. The analysis correlated well with the significant overexpression of the TFs Atf2, Foxf1a, Smad4, Sox4, Sp3 and Stat5a. Finally, we analyzed promoters of regulated genes which where apparently not regulated by c-Myc or other c-Myc targeted TFs and identified overrepresented Oct1, Mzf1, Ppargamma, Plzf, Ets, and HmgIY binding sites when compared against control promoter background.

**Conclusion:**

Our in silico data suggest a model of a transcriptional regulatory network in which different TFs act in concert upon c-Myc overexpression. We determined molecular rules for transcriptional regulation to explain, in part, the carcinogenic effect seen in mice overexpressing the c-Myc oncogene.

## Background

The proto-oncogene c-Myc is highly expressed in many cancer types [1-3] and plays a critical role in regulating cell growth, proliferation, loss of differentiation, and apoptosis [4]. In transgenic mice, targeted overexpression of Myc has been shown to be sufficient to induce cancer [5-7]. In our department, a transgenic mouse model was created which overexpresses c-Myc. The c-Myc overexpression in alveolar epithelium of these mice results in the development of bronchiolo-alveolar carcinoma (BAC) and papillary adenocarcinoma (PLAC). Life expectancies of c-Myc transgenics range between 12–14 months.

The molecular mechanisms by which c-Myc functions to effect tumorigenesis have been the subject of extensive research in the past several decades. c-Myc is a transcription factor, a basic helix-loop-helix leucine zipper protein that dimerizes with Max to bind the DNA sequence 5'-CACGTG-3', known as an E box, and activates transcription [8]. Myc also represses transcription through interaction with Miz-1 or through other elements at core promoters [9]. Furthermore, Brenner et al. [10] suggested that c-Myc may also repress transcription by recruitment of a DNA methyl-transferase corepressor Dnmt3a. DNA methylation is the most important epigenetic modification in mammalian cells and is associated with transcriptional repression. Nevertheless, the mechanisms of transcriptional repression by c-Myc seem not to occur by direct binding of c-Myc to the DNA sequence 5'-CACGTG-3', known as an E box, and are not really well understood.

The pleiotropic effects of c-Myc on tumorigenesis are thought to be mediated by its target genes, because transcriptionally defective Myc alleles have diminished transforming potential [11]. Furthermore, the domain that is required for c-Myc DNA binding, the basic helix-loop-helix zipper domain, is essential for its oncogenic transformation, and c-Myc possesses an N-terminal transactivation domain. Deletions or mutations in this domain result in loss of c-Myc transformation [12]. The transcriptional activation potential of c-Myc, however, does not always correlate with its ability to transform rodent fibroblast cells [13]. Several studies showed that mutations in the Myc box II domain within c-Myc can abrogate its transformation capacity without affecting c-Myc activation of reporter gene constructs [14,15]. These results emphasized the complex and interrelated nature of c-Myc-mediated transformation and highlighted the need to identify specific factors that interact with functionally important domains of the c-Myc oncoproteins.

Despite extensive research, the specific mechanisms by which tumorigenesis are achieved are not well understood. This is largely because a comprehensive list of biologically relevant Myc target genes has not yet been defined and such "transformation" associated genes remain elusive [16]. In order to elucidate Myc targets many different techniques have been employed, such as microarray profiling, serial analysis of gene expression, and chromatin immunoprecipitation [17-25]. To date, > 1,600 genes have been found to be Myc-responsive and stored in the Myc target gene database [26,27], but only a minority of the Myc-responsive genes have been implicated as direct target genes. C-Myc seems to induce a regulatory gene network, which consists of direct and indirect responses. The direct responses also seem to depend on different other transcription factors which either cooperate with or compete against c-Myc. Some of these transcription factors have already been described in the literature [28,29].

In this study, we report a genetic and bioinformatic approach designed to identify regulatory gene networks induced by overexpression of c-Myc in alveolar epithelium of our female transgenic mouse model, resulting in the development of bronchiolo-alveolar carcinoma (BAC) and papillary adenocarcinoma (PLAC). Because the mechanisms of transcriptional repression by c-Myc do not occur by direct binding of c-Myc to E boxes, we restricted our analysis to promoter sequences of induced genes in which the potential c-Myc binding sites can be identified in silico. Thus, we have identified potential direct target genes of c-Myc and propose different transcription factors which either cooperate with or compete against c-Myc. Furthermore, we in silico describe different indirect mechanisms possibly participating in the Myc tumorigenic phenotype. Taken together, we suggest a model of a regulatory gene network in which different TFs act in concert upon overexpression of c-Myc in our transgenic mouse model.

## Results

### Analysis of high-density oligonucleotide microarray data

Global gene expression studies were done with lung tissue stemming from a female mouse transgenic line overexpressing the c-Myc proto-oncogene. The complete data have been deposited in NCBIs Gene Expression Omnibus (GEO) [30] and are accessible through GEO Series accession number GSE10954. The quantitative changes in significantly altered genes were investigated. For the definition of "significantly altered", see the Methods section. According to these criteria, transcription of 469 genes was induced and transcription of 8 genes was repressed in 5 months old animals (data shown in Additional file 1). At this time point the tumor burden was approximately 50%. It must be mentioned here that gene expression profiling by microarrays does not provide information about rates of transcription but measure mRNA abundance which might have been modified by processes such as reduced RNA degradation.

### Validation of microarray data by real time PCR

**Figure 1:**
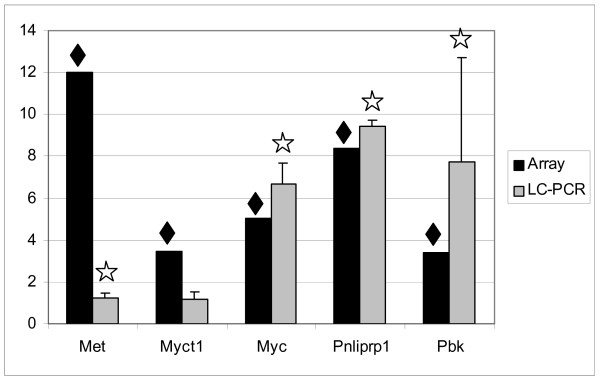
**Validation of microarray data by real time PCR**. Comparison of gene expression of selected genes determined by microarray analysis (black bars) and real time PCR (grey bars; LC: LightCycler^®^). Fold changes are shown on the y-axis. Significant changes of gene expression are indicated either with a diamond for array analysis or with an asterisk for real time PCR.

For the validation of microarray data, five different genes were selected: Met (met proto-oncogene), Myct1 (myc target 1), Myc (myelocytomatosis oncogene), Pnliprp1 (pancreatic lipase related protein 1) and Pbk (PDZ binding kinase). Expression of these genes was alternatively investigated with real time quantitative PCR using the LightCycler^®^. Comparison of fold changes determined by microarray analysis and real time PCR are shown in Figure 1. Statistical significant changes in microarray analysis are indicated by a black diamond. Criteria for significance are described in the methods section. Statistical significant changes in real time PCR are marked with an asterisk, which is based on a paired two-tailed student t-Test. The results were considered significant when the p-value was less than 0.05. As shown in Figure 1 there was basic agreement between the two platforms. The fold changes of Met, however, differ strongly between microarray analysis and real time PCR. This phenomenon can be observed sometimes with the validation of microarray data by real time PCR: microarray analysis shows strong up regulation whereas PCR indicates a very low fold change like 1.5 or less. Here, the reason might be the low average intensity value of 40.01 combined with its high standard deviation of 67.12% for Met in the microarray experiments of non-transgenic animals. Notably, the average standard deviation of all significantly regulated genes of this study amounts 23.81%. Together with a high and stable average intensity value of 480 combined with its low standard deviation of 16.83% for Met in the microarray experiments of c-myc-transgenic animals the corresponding fold change appears higher than it might be in fact. Furthermore, we did not compare gene expression on identical sequences. Hence, we can not exclude the possibility that transcript expression differs on the basis of the different sequences (primers and amplification products) employed.

### Promoter sequence analysis of genes induced by overexpression of c-Myc

**Figure 2:**
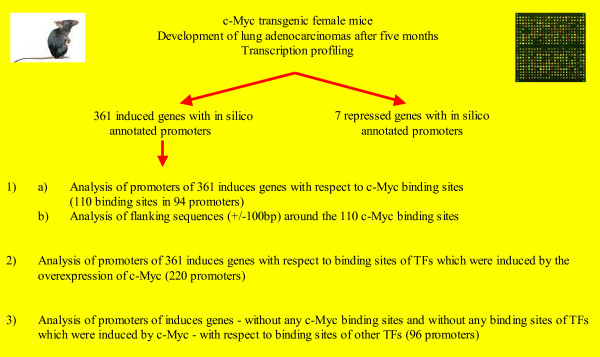
Flow chart of our in silico strategy used to elucidate the c-Myc regulatory network.

A flowchart of our in silico strategy used to elucidate the c-Myc regulatory network is depicted in Figure 2.

#### 1) Analysis of promoters of 361 induced genes with respect to c-Myc binding sites

By using their RefSeq annotation, 361 promoter sequences could be extracted from the UCSC Genome Browser for the 469 upregulated genes. Furthermore, promoters of 100 genes which were not regulated at all were extracted (the list of non-regulated genes was prepared after applying criteria according to the Methods section and was included in Additional file 2). Both sequence sets were analyzed using TRANSFAC^® ^Professional rel. 8.3 integrated tool MATCH^® ^by using the matrices V$EBOX_Q6_01 (cut-off core similarity: 1.00, matrix similarity: 0.99), V$MYC_Q2 (cut-off core similarity: 1.00, matrix similarity: 0.99), and V$MYCMAX_B (cut-off core similarity: 0.75, matrix similarity: 0.96). The results of these analyses including positions and sequences of the corresponding binding sites are given in Additional file 3. Altogether, 110 different c-Myc binding sites were found in 94 different promoters, which partly were recognized by different matrices. Table 1 gives the 94 genes which are putatively directly regulated by c-Myc and the corresponding biological process they are involved in. In this table, the 15 targets stored already in the Myc Target Database are marked bold. Moreover, the number of c-Myc binding sites identified in the promoter set including promoters of induced genes was compared to the number of c-Myc binding sites identified in the control promoter set. The corresponding calculated fold occurrences of binding sites and the significance (p-value) of the occurrence values are given in Table 2. The fold occurrences describe the number of c-Myc binding sites detected as a ratio with regard to the number of gene promoters analyzed. Here, the fold occurrence of the non-regulated gene promoters is set to 1. For all matrices used the fold occurrences of the c-Myc binding sites of the analyzed promoters in total are increased in comparison to the fold occurrences of the c-Myc binding sites of the control gene promoters in total. Furthermore, the significance of the occurrence values is high. This result indicates direct regulation by c-Myc of many of the genes analyzed.

**Table 1:** Putative direct c-Myc targets: biological processes

**Probe Set ID**	**Gene Symbol**	**Gene Title**	**Biological process**
1420821_at	Sgpp1	sphingosine-1-phosphate phosphatase 1	apoptosis
1428480_at	Cdca8	cell division cycle associated 8	cell cycle
1435306_a_at	Kif11	kinesin family member 11	cell cycle
1423903_at	Pvr	poliovirus receptor	cell-cell adhesion
1422889_at	Pcdh18	protocadherin 18	cell-cell adhesion
1427489_at	Itga8	integrin alpha 8	cell-matrix adhesion
1419717_at	Sema3e	sema domain, immunoglobulin domain (Ig), short basic domain, secreted, (semaphorin) 3E	development
1448595_a_at	Bex1	brain expressed gene 1	development
1422198_a_at	**Shmt1**	serine hydroxymethyltransferase 1 (soluble)	amino acid metabolism
1418483_a_at	Ggta1	glycoprotein galactosyltransferase alpha 1, 3	carbohydrate metabolism
1421879_at	Mtmr1	myotubularin related protein 1	lipid metabolism
1426575_at	Sgms1	sphingomyelin synthase 1	lipid metabolism
1445103_at	Tmem23	Transmembrane protein 23	lipid metabolism
1417300_at	Smpdl3b	sphingomyelin phosphodiesterase, acid-like 3B	lipid metabolism
1421957_a_at	**Pcyt1a**	phosphate cytidylyltransferase 1, choline, alpha isoform	lipid metabolism
1422702_at	Azin1	antizyme inhibitor 1	polyamine metabolism
1417190_at	Pbef1	pre-B-cell colony-enhancing factor 1	NAD biosynthetic process
1423088_at	Tmod3	tropomodulin 3	negative regulation of cell motility
1420847_a_at	Fgfr2	fibroblast growth factor receptor 2	proliferation
1415691_at	Dlg1	discs, large homolog 1 (Drosophila)	proliferation
1422966_a_at	**Tfrc**	transferrin receptor	proliferation
1420924_at	**Timp2**	tissue inhibitor of metalloproteinase 2	proliferation
1420020_at	Suz12	suppressor of zeste 12 homolog (Drosophila)	proliferation
1416657_at	**Akt1**	thymoma viral proto-oncogene 1	proliferation
1420852_a_at	B3gnt2	UDP-GlcNAc:betaGal beta-1,3-N-acetylglucosaminyltransferase 2	protein amino acid glycosylation
1426342_at	Stt3b	STT3, subunit of the oligosaccharyltransferase complex, homolog B (S. cerevisiae)	protein amino acid glycosylation
1425581_s_at	Galnt7	UDP-N-acetyl-alpha-D-galactosamine: polypeptide N-acetylgalactosaminyltransferase 7	protein amino acid glycosylation
1415692_s_at	**Canx**	calnexin	protein folding
1429502_at	Stch	stress 70 protein chaperone, microsome-associated, human homolog	protein folding
1427074_at	Pcmtd2	protein-L-isoaspartate (D-aspartate) O-methyltransferase domain containing 2	protein modification
1426721_s_at	Tiparp	TCDD-inducible poly(ADP-ribose) polymerase	protein modification
1439151_at	Msrb3	methionine sulfoxide reductase B3	protein repair
1431361_at	Prcp	prolylcarboxypeptidase (angiotensinase C)	proteolysis
1421857_at	Adam17	a disintegrin and metallopeptidase domain 17	proteolysis
1422528_a_at	**Zfp36l1**	zinc finger protein 36, C3H type-like 1	regulation of mRNA stability
1420975_at	**Baz1b**	bromodomain adjacent to zinc finger domain, 1B	regulation of transcription, DNA-dependent
1421162_a_at	Nfia	nuclear factor I/A	regulation of transcription, DNA-dependent
1423773_at	Gpbp1	GC-rich promoter binding protein 1	regulation of transcription, DNA-dependent
1423441_at	Tfb2m	transcription factor B2, mitochondrial	regulation of transcription, DNA-dependent
1422864_at	Runx1	runt related transcription factor 1	regulation of transcription, DNA-dependent
1416018_at	Dr1	down-regulator of transcription 1	regulation of transcription, DNA-dependent
1418280_at	Klf6	Kruppel-like factor 6	regulation of transcription, DNA-dependent
1425465_a_at	Senp2	SUMO/sentrin specific peptidase 2	regulation of transcription, DNA-dependent
1421908_a_at	**Tcf12**	transcription factor 12	regulation of transcription, DNA-dependent
1434643_at	Tbl1x	transducin (beta)-like 1 X-linked	regulation of transcription, DNA-dependent
1426531_at	Zmynd11	zinc finger, MYND domain containing 11	regulation of transcription, DNA-dependent
1419976_s_at	Nfatc3	nuclear factor of activated T-cells, cytoplasmic, calcineurin-dependent 3	regulation of transcription, DNA-dependent
1432143_a_at	**Hbp1**	high mobility group box transcription factor 1	regulation of transcription, DNA-dependent
1427418_a_at	**Hif1a**	hypoxia inducible factor 1, alpha subunit	regulation of transcription, DNA-dependent
1421835_at	Mtap7	microtubule-associated protein 7	response to osmotic stress
1415996_at	Txnip	thioredoxin interacting protein	response to oxidative stress
1416467_at	Ddx3x	DEAD/H (Asp-Glu-Ala-Asp/His) box polypeptide 3, X-linked	RNA helicase activity
1415807_s_at	**Sfrs2**	splicing factor, arginine/serine-rich 2 (SC-35)	RNA splicing
1425625_at	Il13ra1	interleukin 13 receptor, alpha 1	signal transduction
1426892_at	Utrn	utrophin	signal transduction
1433706_a_at	Ptplad1	protein tyrosine phosphatase-like A domain containing 1	signal transduction
1424947_at	Dync1li1	dynein cytoplasmic 1 light intermediate chain 1	signal transduction
1425538_x_at	Ceacam1	CEA-related cell adhesion molecule 1	signal transduction
1420918_at	Sgk3	serum/glucocorticoid regulated kinase 3	signal transduction
1437295_at	Pkn2	protein kinase N2	signal transduction
1416504_at	Ulk1	Unc-51 like kinase 1 (C. elegans)	signal transduction
1418489_a_at	Calcrl	calcitonin receptor-like	signal transduction
1421239_at	Il6st	interleukin 6 signal transducer	signal transduction
1420814_at	Gdi2	guanosine diphosphate (GDP) dissociation inhibitor 2	signal transduction
1420696_at	Sema3c	sema domain, immunoglobulin domain (Ig), short basic domain, secreted, (semaphorin) 3C	signal transduction
1418022_at	Narg1	NMDA receptor-regulated gene 1	transcription
1420307_a_at	Pitpnb	phosphatidylinositol transfer protein, beta	transport
1419970_at	Slc35a5	solute carrier family 35, member A5	transport carbohydrate
1421839_at	Abca1	ATP-binding cassette, sub-family A (ABC1), member 1	transport cholesterol
1417622_at	**Slc12a2**	solute carrier family 12, member 2	transport ion
1418257_at	Slc12a7	solute carrier family 12, member 7	transport ion
1416832_at	Slc39a8	solute carrier family 39 (metal ion transporter), member 8	transport metal ion
1426712_at	Slc6a15	solute carrier family 6 (neurotransmitter transporter), member 15	transport neurotransmitter
1421641_at	Slc6a2	solute carrier family 6 (neurotransmitter transporter, noradrenalin), member 2	transport neurotransmitter
1421167_at	Atp11a	ATPase, class VI, type 11A	transport phospholipid
1423597_at	Atp8a1	ATPase, aminophospholipid transporter (APLT), class I, type 8A, member 1	transport phospholipid
1428065_at	Slc44a2	solute carrier family 44, member 2	transport protein
1416375_at	Ap3m1	adaptor-related protein complex 3, mu 1 subunit	transport protein
1436508_at	2410014A08Rik	RIKEN cDNA 2410014A08 gene	transport protein
1416189_a_at	Sec61a1	Sec61 alpha 1 subunit (S. cerevisiae)	transport protein
1426775_s_at	**Scamp1**	secretory carrier membrane protein 1	transport protein
1420867_at	Tmed2	transmembrane emp24 domain trafficking protein 2	transport protein
1421955_a_at	Nedd4	neural precursor cell expressed, developmentally down-regulted gene 4	ubiquitin cycle
1416680_at	Ube3a	ubiquitin protein ligase E3A	ubiquitin cycle
1426495_at	2410042D21Rik	RIKEN cDNA 2410042D21 gene	?
1419152_at	2810417H13Rik	RIKEN cDNA 2810417H13 gene	?
1421603_a_at	Ceacam2	CEA-related cell adhesion molecule 2	?
1417821_at	D17H6S56E-5	DNA segment, Chr 17, human D6S56E 5	?
1423557_at	Ifngr2	interferon gamma receptor 2	?
1421064_at	Mpp5	membrane protein, palmitoylated 5 (MAGUK p55 subfamily member 5)	?
1421814_at	**Msn**	moesin	?
1423479_at	Nol11	nucleolar protein 11	?
1426806_at	Obfc2a	oligonucleotide/oligosaccharide-binding fold containing 2A	?
1424778_at	Reep3	receptor accessory protein 3	?

The table gives the 94 genes which are putatively directly regulated by c-Myc and the corresponding biological processes they are involved in. In this table, the 15 targets stored already in the Myc Target Database are marked bold.

**Table 2:** Fold occurrences of c-Myc binding sites in the promoters of induced genes.

**Matrices applied in the analysis of promoter sequences of 361 c-Myc-regulated genes**	**Number of binding sites found in all promoter sequences**	**Fold occurrence of binding sites**	**Significance (p-value)**
V$EBOX_Q6_01	28	1.55	0.006492733
V$MYC_Q2	115	1.10	0.022316242
V$MYCMAX_B	52	1.31	0.008330239

The occurrence value of c-Myc binding sites in the promoters of control genes is set to 1. Significance of the representation value of c-Myc binding sites in the promoters of induced genes is measured by the p-value derived from the binomial distribution.

#### 2) Analysis of flanking sequences (+/- 100 bp) around the 110 c-Myc binding sites

For this analysis, we extracted the 110 c-Myc binding sites including the flanking sequences (100 bp flanking the 5 bp core sequence to both sides (= 205 bp)). We further randomly extracted the same number of 205 bp sequences from the control promoters which were not regulated at all (the list of 205 bp sequences of non-regulated genes was included in Additional file 4). Both sequence sets were analyzed using TRANSFAC^® ^Professional rel. 8.3 integrated tool MATCH^® ^by using the matrix profile "vertebrates_minSUM_highQual". An extract of the results of these analyses including the numbers of transcription factor binding sites in the corresponding promoter sets, the following fold occurrence of a given TF, and the significance (p-value) of the occurrence values are listed in Table 3. The complete result of this analysis is given in Additional file 5. According to Table 3, the putative c-Myc binding sites in the 110 analyzed sequences have been identified by six different matrices included in the profile used (V$MYC_Q2, V$MYCMAX_01, V$MYCMAX_02, V$MYCMAX_B, V$MAX_01, and EBOX_Q6_01). The 110 different putative c-Myc binding sites were recognized partly by different matrices. As expected the significance of the occurrence values for each c-Myc matrice is high, respectively. Furthermore, the number of hits of different matrices for the transcription factors E2F, AP2, ZF5, and ZIC3 clearly shows a highly significant overrepresentation in comparison to control sequences which contain nearly no c-Myc binding sites.

**Table 3:** Analysis of flanking sequences (+/- 100 bp) around the 110 c-Myc binding sites

**Matrix**	**Hits in sequences (205 bp) of induced promoters**	**Hits in sequences (205 bp) of control promoters**	**Fold-Occurrence**	**P-Wert**
V$MYC_Q2	96	7	13.71	1.04731E-98
V$MYCMAX_01	16	4	4.00	2.00649E-06
V$MYCMAX_02	10	1	10.00	7.25446E-08
V$MYCMAX_B	18	2	9.00	1.73837E-12
V$MAX_01	34	6	5.67	4.50618E-17
V$EBOX_Q6_01	25	2	12.50	2.36615E-20
V$E2F_Q2	50	21	2.38	2.08371E-10
V$E2F1_Q3	22	7	3.14	1.12327E-06
V$E2F1_Q3_01	11	3	3.67	0.000171353
V$E2F1_Q6	11	5	2.20	0.007294131
V$AP2_Q6	17	7	2.43	0.000392586
V$AP2_Q6_01	14	5	2.80	0.000339388
V$AP2ALPHA_03	30	8	3.75	1.41269E-10
V$AP2GAMMA_01	21	2	10.50	1.04239E-15
V$ZF5_B	85	37	2.30	7.71777E-21
V$ZIC3_01	22	10	2.20	0.000213134

The TRANSFAC identifier of the respective matrix, the number of hits in the 110 different sequences (205 bp) of induced gene promoters, the number of hits in the 110 different sequences (205 bp) of control (non-regulated) gene promoters, and the corresponding fold occurrences are given in this table. The occurrence value of the respective binding site in the promoters of control genes is set to 1. Significance of the representation value of binding sites in flanking sequences (+/- 100 bp) around the 110 c-Myc binding sites in the promoters of induced genes is measured by the p-value derived from the binomial distribution.

**Figure 3:**
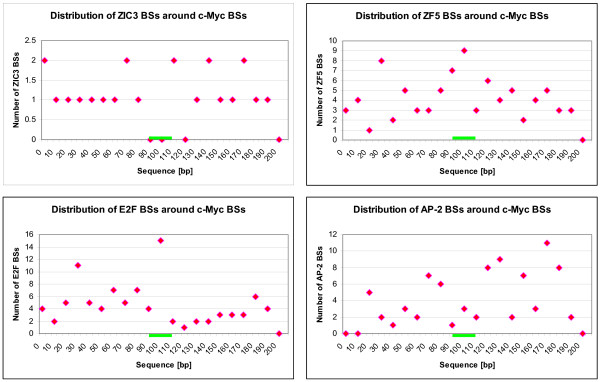
**Distribution of overrepresented TFs around the c-Myc binding sites (+/- 100 bp)**. Localization of binding sites (BSs) for the respective transcription factor (TF) within the flanking sequences around the c-Myc BSs. The position in bp within the whole extracted and analyzed sequence is given on the x-axis. The core sequences of c-Myc BSs are located at bp 100 to 105 and are marked by the green lines. The number of identified BSs are shown on the y-axis.

This might mean that these TFs bind in the nearest neighborhood to c-Myc in order to either cooperate with or compete against c-Myc. The distribution of these TFs around the c-Myc binding sites is shown in Figure 3. Here, the diagrams show that AP2 and ZIC3 do not or nearly not bind to the same site as c-Myc does, whereas E2F and ZF5 in some cases seem to bind to the same site as c-Myc.

#### 3) Analysis of promoters of 361 induced genes with respect to binding sites of TFs which were transcriptionally induced by overexpression of c-Myc

According to GeneOntology 36 of 477 deregulated genes possess transcription factor activity or transcription regulator activity (Additional file 6). In the database TRANSFAC^® ^Professional rel. 8.3, however, positional weight matrices are available only for the 6 transcription factors Atf2, Foxf1a, Smad4, Sox4, Sp3, and Stat5a, which were upregulated by overexpression of c-Myc.

Next, we analyzed the 361 promoter sequences out of 469 upregulated genes which are available in the UCSC Genome Browser, using the TRANSFAC^® ^Professional rel. 8.3 integrated tool MATCH^® ^by applying the matrices V$CREBATF_Q6 (cut-off core similarity: 1.00, matrix similarity: 0.98), V$HFH8_01 (cut-off core similarity: 1.00, matrix similarity: 0.97), V$SMAD-4 (cut-off core similarity: 0.95, matrix similarity: 0.88), V$SOX_Q6 (cut-off core similarity: 1.00, matrix similarity: 0.88), V$STAT5A_03 (cut-off core similarity: 1.00, matrix similarity: 1.00), V$STAT5A_04 (cut-off core similarity: 1.00, matrix similarity: 1.00), V$STAT5A_01 (cut-off core similarity: 1.00, matrix similarity: 0.98), V$STAT5A_02 (cut-off core similarity: 1.00, matrix similarity: 0.83), and V$SP3_Q3 (cut-off core similarity: 0.90, matrix similarity: 0.91). The results of this analysis including positions and sequences of the corresponding binding sites are given in Additional file 7. Altogether, 368 putative binding sites were found in 220 different promoters. 115 binding sites for Atf2 (V$CREBATF_Q6) in 73 different promoters were identified, 44 binding sites for Foxf1a (V$HFH8_01) in 42 different promoters, 53 binding sites for Smad4 (V$SMAD_4) in 47 different promoters, 33 binding sites for Sox4 (V$SOX_Q6) in 31 different promoters, 82 binding sites for Stat5a (V$STAT5A_01, V$STAT5A_02, V$STAT5A_03, V$STAT5A_04) in 71 different promoters, and 41 binding sites for SP3 (V$SP3_Q3) in 39 different promoters. They are listed in Additional file 8. 46 out of the 220 promoters possessing a binding site of a transcription factor whose transcription was induced by c-Myc possess also a c-Myc binding site (see Additional file 9).

#### 4) Analysis of promoters of induced genes – without any c-Myc binding sites and without any binding sites of TFs which were induced by c-Myc – with respect to binding sites of other TFs

**Figure 4:**
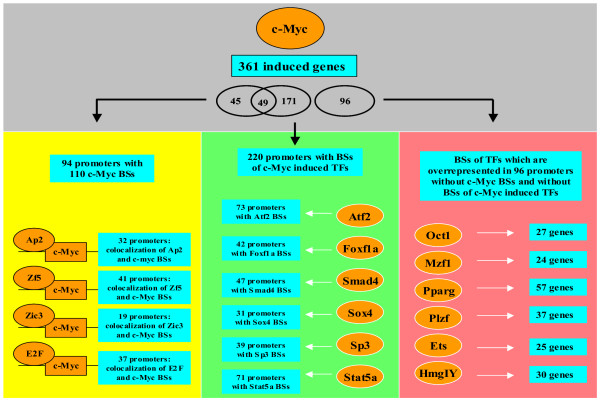
**Coordinate events which resulted in gene regulation in response to c-Myc overexpression**. 1) c-Myc putatively binds to 110 binding sites in 94 different promoters of induced genes. Binding sites (BSs) of four different transcription factors (TFs) have been shown to be overrepresented in the direct neighborhood of c-Myc binding sites. They either cooperate with or compete againstc-Myc. 2) Some of the genes induced by c-Myc overexpression code for transcription factors. These transcription factors constitute the basis of a regulatory gene network that again influences the expression of different genes. Taken collectively, 220 different promoters of induced genes possess binding sites for those induced transcription factors, matrices of which areavailable. 3) Other transcription factors which could be part of the c-Myc regulatory gene network are those which might be activated on the protein level through the overexpression of c-Myc.

The 96 promoters of genes induced by overexpression of c-Myc which possess neither a putative c-Myc binding site nor a binding site of a transcription factor which was transcriptionally induced by c-Myc were analyzed using TRANSFAC^® ^Professional rel. 8.3 integrated tool MATCH^® ^by applying the matrix profile "vertebrates_minSUM_highQual". We further performed the same analysis using control promoters which were not regulated at all (Additional file 2). An extract of the results of these analyses including the numbers of transcription factor binding sites in the corresponding promoter sets and the resulting fold occurrence of a given TF are listed in Table 4. According to this table, the different matrices for the transcription factors Oct1, Mzf1, Pparg, Plzf, Ets, and HmgIY provide more than 30 hits. This table clearly shows an overrepresentation in comparison to control sequences. We found 36 putative Oct1 binding sites in 27 promoters, 37 putative Mzf1 binding sites in 24 promoters, 131 putative Pparg binding sites in 57 promoters, 47 putative Plzf binding sites in 37 promoters, 42 putative Ets binding sites in 25 promoters, and 46 putative HmgIY binding sites in 30 promoters. They are listed in Additional file 8. A summary of all results is depicted in Figure 4.

**Table 4:** Analysis of promoters of induced genes – without any c-Myc binding sites and without any binding sites of TFs which were induced by c-Myc – with respect to binding sites of other TFs

**Matrix**	**Number of binding sites in promoters of 96 induced genes**	**Number of binding sites in promoters of 100 non-regulated genes**	**Fold occurrence**	**Significance (p-value)**
V$HNF4_01	6	1	6.25	0.000375206
V$CREL_01	10	2	5.21	2.03259E-05
V$NFKAPPAB65_01	5	1	5.21	0.002449147
V$NFKB_Q6_01	5	1	5.21	0.002449147
V$PPARG_01	11	4	2.86	0.001151136
V$NFKB_C	5	2	2.6	0.031112513
V$P53_DECAMER_Q2	10	4	2.6	0.003533721
V$COUPTF_Q6	12	5	2.5	0.002051505
V$ELF1_Q6	12	5	2.5	0.002051505
V$IRF_Q6_01	26	11	2.46	7.01224E-06
V$P53_02	7	3	2.43	0.017329258
V$NF1_Q6	18	8	2.34	0.000369053
V$PPAR_DR1_Q2	11	5	2.29	0.005502861
V$CEBPGAMMA_Q6	19	9	2.2	0.000532298
V$AP1_Q4	10	5	2.08	0.013373232
V$NF1_Q6_01	10	5	2.08	0.013373232
V$E2F1DP2_01	8	4	2.08	0.023927388
V$E2F4DP2_01	8	4	2.08	0.023927388
V$AP1_Q2	11	6	1.91	0.016631957
V$AR_01	11	6	1.91	0.016631957
V$OCT1_03	36	21	1.79	9.18715E-05
V$HIC1_03	12	7	1.79	0.019471252
V$HNF4_01_B	12	7	1.79	0.019471252
V$PLZF_02	47	28	1.75	6.68834E-06
V$ETS_Q6	20	12	1.74	0.005001828
V$FREAC4_01	10	6	1.74	0.033328378
V$XFD1_01	10	6	1.74	0.033328378
V$OCT1_06	13	8	1.69	0.021905095
V$ETS_Q4	22	14	1.64	0.006219504
V$CAAT_01	14	9	1.62	0.023973145
V$CDPCR1_01	12	8	1.56	0.038985854
V$E47_02	18	12	1.56	0.017018836
V$P53_01	6	4	1.56	0.096354785
V$STAT_Q6	12	8	1.56	0.038985854
V$MZF1_02	37	25	1.54	0.001169513
V$PPARG_02	79	64	1.52	0.001699828
V$HMGIY_Q3	46	32	1.5	0.000431589

The TRANSFAC identifier of the respective matrix, the number of hits in the 96 induced gene promoters, the number of hits in the 100 non-regulated control gene promoters, and the corresponding fold occurrences are given in this table. The occurrence value of the respective binding site in the promoters of control genes is set to 1. Significance of the representation value of TF binding sites in the promoters is measured by the p-value derived from the binomial distribution.

## Discussion

Transcription profiling studies have identified many target genes activated or repressed by c-Myc in various animal and human cells or cell lines. The number of experimentally validated c-Myc targets is rapidly expanding thanks to the use of high-throughput methods [19,31-33]. Two recent studies suggest that the potential list of c-Myc targets could be much larger than what was previously anticipated [22,31]. Moreover, Chen et al. [32] suggest the existence of a significant tissue-specific component in the response of many c-Myc target genes. Gene expression studies alone, however, cannot discriminate between direct and indirect targets of c-Myc action, although network-based interference of direct action has been proposed [31]. Furthermore, gene expression studies alone can identify neither transcription factor activations or repressions on the protein level nor transcriptional cooperation and competition of different transcription factors involved in the corresponding regulatory network. Analysis of promoters of regulated genes resulting from gene expression studies, however, may provide indications in these directions.

Thus, using positional weight matrices (PWMs), which is the most widely used method for recognition of transcription factor binding sites [34,35], we analyzed promoters of genes which were induced by overexpression of c-Myc in alveolar epithelium of our female transgenic mouse model resulting in the development of bronchiolo-alveolar carcinoma (BAC) and papillary adenocarcinoma (PLAC), in order to elucidate the c-Myc transcriptional regulatory network consisting of direct and indirect mechanisms possibly participating in the Myc tumorigenic phenotype. We wish to point out that the c-Myc transcriptional regulatory network analyzed in other tissues might be different from the network described in this study. Indeed, an analysis of 89 genes whose promoters (1000 bp upstream of the TSS) possess at least one experimentally determined high-quality Myc binding locus on human P493 B cells [29] provided no overlap with promoters of genes in mouse lung adenocarcinoma reported in the present study.

By applying three different weight matrices for recognition of c-Myc binding sites, we predicted 94 putative c-Myc targets among the genes presented on Affymetrix platform GeneChip^® ^Mouse Genome 430 2.0. This list contains 15 targets stored in the Myc Target Database, whereas 79 genes are putative novel targets. Whether they are real targets remains to be elucidated. The functional categories of these 94 putative c-Myc targets revealed that various cellular processes like transcriptional regulation, protein modification and transport, cell cycle control/proliferation, metabolism, and signal transduction are putatively directly regulated by c-Myc. These findings correlate well with expectations based on the biology of c-Myc.

In higher organisms transcription factors usually operate in combination with other transcription factors bound in direct neighborhood in promoter sequences to influence gene transcription. Up to now, less is known about transcription factors (TFs) collaborating with c-Myc. Previously, Elkon et al. [36] reported in silico identified transcriptional regulators associated with c-Myc activity in human Burkitt's lymphoma cells and this included overrepresentation of binding sites of the transcription factors ETF, SP1, Nrf-1, NF-Y, CREB, Egr-1, Elk-1, E2F and AhR/Arnt in c-Myc target genes. The extracted and analyzed promoters spanned 1000 bp upstream to 200 bp downstream of the transcription start sites of the corresponding genes.

In the present study, we analyzed exclusively the flanking sequences around the in silico identified c-Myc binding sites by use of all available positional weight matrices in the TRANSFAC database. Especially binding sites of the transcription factors E2F, AP2, ZF5, and ZIC3 were found to be significantly enriched from 2.2- to 10-fold over control promoter background. The poor concordance of our results and those of Elkon et al. [36] might be due to different reasons: We analyzed different species, different tissues, and different lengths of analyzed sequences and therefore, possibly different distances from c-Myc binding sites.

**Figure 5:**
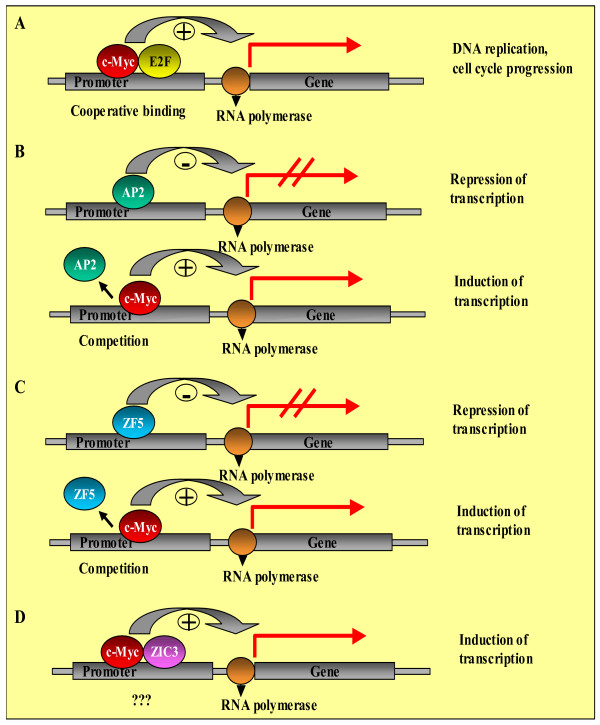
Putative direct actions of c-Myc in part in cooperation or competition with other transcription factors.

Notably, both studies identified E2F to be a transcriptional regulator associated with c-Myc. Like c-Myc, E2F also controls cell cycle progression and DNA replication [37]. Thus, deregulation of c-Myc could potentially lead to uncontrolled cell cycle progression through a functional link with E2F, as proposed also by Zeller et al. [29]. The authors supposed that high c-Myc expression leads to increased E2F activity by upregulating genes involved in cell cycle control. The cooperative binding of Myc and E2F followed by transcriptional activation of key downstream targets leads to an increase in DNA replication and cell cycle progression (Figure 5). Here, by using four different matrices for E2F, we found E2F binding sites in the direct neighborhood of c-Myc binding sites (maximum distance from c-Myc binding sites was 100 bps) in 37 sequences out of 110 sequences possessing a c-Myc binding site. Depending on the matrix used, they are 2.2- to 3.7-fold enriched over the control promoter background. Furthermore, the network relationships between c-Myc and E2F are also obvious through the identification of functional E2F binding sites in the c-Myc [38] and in the E2F promoter [39] as well as the identification of E2F as a c-Myc target gene [26].

By using four different matrices for AP2, we also found AP2 binding sites in the direct neighborhood of c-Myc binding sites in 32 sequences out of 110 sequences possessing a c-Myc binding site. Depending on the matrix applied, they are 2.4- to 10.5-fold enriched over the control promoter background. In 2006, Zeller et al. already identified AP2 to be significantly enriched in cis-regulatory modules with c-Myc [29]. The AP2 family of transcription factors plays a broad range of roles in cell growth, tissue morphogenesis, and cancers. One of the mechanisms by which the AP2 family fulfills their roles is to activate or suppress various downstream target genes at transcriptional levels. A number of studies demonstrated that AP2-interacting proteins can affect the transcription of AP2 downstream targets by modulating the transcriptional activity of AP2. In fact, several AP2-interacting partners have been identified, such as YY1, RB, and c-Myc [28,40,41]. Thus, AP2 is a known c-Myc partner. In 1995, Gaubatz et al. [28] showed that AP2 acts as an inhibitor of Myc-mediated transactivation, a function that is mediated both by competition of AP2 with binding of Myc or Max and by direct protein-protein interaction of AP2 with the BR/HLH/LZ domain of the Myc protein. Here, in the promoters of which both binding sites – c-Myc and AP2 – were found in the direct neighborhood, the overrepresented transcription factor c-Myc might have induced the corresponding gene transcription, whereas under normal conditions AP2 might be able to inhibit this transactivation (Figure 5).

We also found ZF5 binding sites in the direct neighborhood of c-Myc binding sites in 41 sequences out of 110 sequences possessing a c-Myc binding site. They are 2.3-fold enriched over the control promoter background. ZF5 is a ubiquitously expressed protein originally identified by its ability to bind and repress the murine c-Myc promoter [42]. It contains an N-terminal POZ domain, which is a conserved protein-protein interface that recruits cofactors to modulate transcription [43]. ZF5 mediates both transcriptional activation and repression of cellular and viral promoters [42-44]. Here, in the promoters of which both binding sites – c-Myc and ZF5 – were found in the direct neighborhood, the overrepresented transcription factor c-Myc might have induced the corresponding gene transcription, whereas under normal conditions ZF5 might be able to competitively inhibit this transactivation and further inhibit the transcription of the c-Myc gene (Figure 5).

Furthermore, we found ZIC3 binding sites in the direct neighborhood of c-Myc binding sites in 19 sequences out of 110 sequences possessing a c-Myc binding site. They are 2.2-fold enriched over the control promoter background. ZIC3 is a developmental specific zinc finger transcription factor defining early embryo patterning [45]. Although Zic3 expression has been implicated in embryonic development, however, a detailed understanding is still missing of what regulates Zic3 expression and what the downstream effectors of Zic3 are. Until now, there is no indication of connectivity of Zic3 and c-Myc, with one exception: in 2006, Zeller et al. found that the Zic3 binding motifs are significantly enriched in c-Myc-repressed genes after a genome-wide characterization of direct c-Myc binding targets in a model of human B lymphoid tumor using ChiP coupled with pair-end ditag sequencing analysis (ChiP-PET). Here, however, we found Zic3 binding motifs to be significantly enriched in c-Myc-induced genes after overexpression of c-Myc in alveolar epithelium of our female transgenic mouse model (Figure 5). To elucidate the biological significance of this observation further studies are necessary.

**Figure 6:**
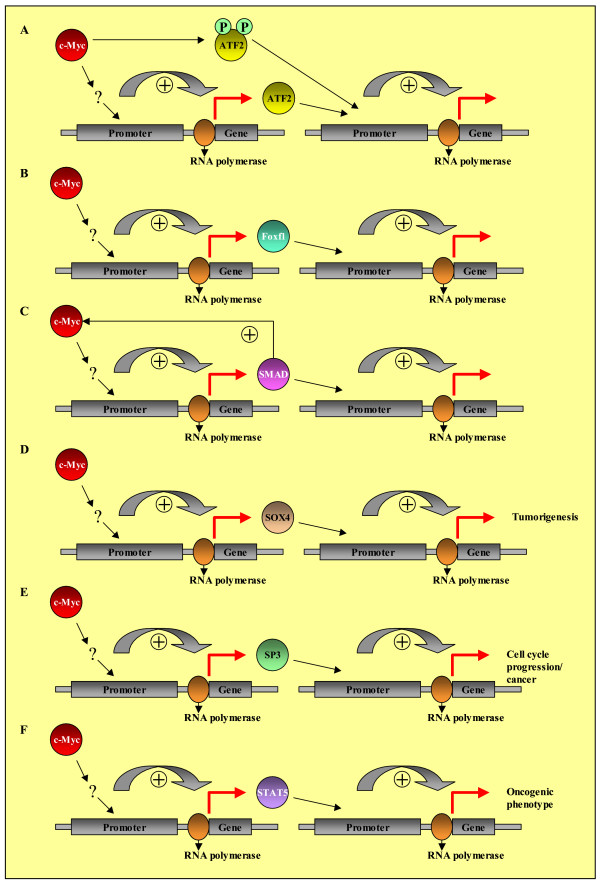
Putative indirect actions of c-Myc, mediated by transcription factors induced by the overexpression of c-Myc.

As mentioned above, gene expression studies alone cannot discriminate between direct and indirect targets of c-Myc action. Nevertheless, with overexpression of c-Myc in alveolar epithelium of our female transgenic mouse model resulting in the development of bronchiolo-alveolar carcinoma (BAC) and papillary adenocarcinoma (PLAC) 36 of 477 deregulated genes possess transcription factor activity or transcription regulator activity. These transcription factors mediate the indirect actions of c-Myc. By using the corresponding available Positional Weight Matrices from TRANSFAC (Atf2, Foxf1a, Smad4, Sox4, Sp3, and Stat5a) for the analysis of the 477 deregulated genes, many putative indirect targets of c-Myc action could be identified. In 73 promoters at least one binding site for ATF2 has been identified. ATF2 belongs to the basic region leucine zipper (bZIP) family of transcription factors and is an important member of activating protein 1 (AP-1) [46]. ATF2 functions either as a homodimer or as a heterodimer with other members of the ATF family as well as other bZIP proteins, to bind to specific DNA sequences and activate gene expression. One major role of ATF2 is to regulate the response of cells to stress signals [47,48]. Furthermore, in 2001, Miethe J et al. [49] identified a crosstalk between Myc and activating transcription factor 2 (ATF2): Myc prolongs the half-life of ATF2 and causes increased phosphorylation of ATF2 at sites that have been shown to be crucial for the regulation of ATF2 activity [49]. Thus, ATF2 is activated by c-Myc on the protein level. Here, we show a novel mechanism for gene activation by c-Myc: the transcriptional activation of the transcription factor ATF2, which in turn putatively activates the transcription of 36 genes (Figure 6). Additionally, Tamura et al. also demonstrated an interaction between ATF2 and c-Myc in rat fibroblasts by affinity chromatography and co-immunoprecipitation [50].

The members of the forkhead box (Fox) family of transcription factors play important roles in regulating transcription of genes involved in cellular proliferation, differentiation, and metabolic homeostasis [51]. Foxf1 RNA is expressed at mesenchymal-epithelial interfaces involved in lung and gut morphogenesis [52]. In the adult mouse, Foxf1 RNA is detected in smooth muscle layers of pulmonary bronchioles, lamina propria of the stomach and the intestine, and in alveolar endothelial cells. Foxf1 is further essential for normal lung repair and endothelial cell survival in response to pulmonary cell injury [53]. Here, we demonstrated transcriptional activation of the Foxf1 gene by overexpression of c-Myc and thus an indirect c-Myc action (Figure 6). Foxf1 putatively activates the transcription of 42 genes.

Negative regulation of c-Myc expression by TGF-β is well established and is a key mechanism through which TGF-β causes G_1 _arrest and inhibition of cell proliferation in epithelial cells. Three studies identified the TIE sequence in the c-Myc promoter as mediating the TGF-β effect on c-Myc expression. A complex of Smad3-Smad4, E2F4/5, DP1, and p107 binds to the TIE in response to TGF-β to inhibit transcription of c-Myc [54]. This Smad-dependent repression of c-Myc expression was previously the only known function of Smad4 in the regulation of c- Myc. Data presented by Lim SK and Hoffmann FM [55] provide evidence that Smad4 can also function as a positive regulator of c- Myc expression in the absence of TGF-β signaling. Here, in turn, we identified the ability of c-Myc to act as a positive regulator of Smad4 expression (indirect or direct). Smad4 again mediates the indirect effects of c-Myc (Figure 6).

The SOX4 gene is highly expressed in human breast cancer cell lines, colon cancer cell lines, hepatocarcinoma, medulloblastomas, and adenoid cystic carcinomas [56-58]. SOX-4 was also shown to be highly and differentially expressed in a substantial fraction of small-cell lung carcinoma (SCLC) samples and in a pool of primary lung adenocarcinoma samples, with very low levels of expression in a number of normal essential tissues. Notably, evidence has been presented to suggest that SOX-4 may be involved in tumorigenesis [59,60]. Here, we identified the ability of c-Myc to act as an indirect positive regulator of SOX-4 expression. SOX-4 again mediates the indirect effects of c-Myc (Figure 6).

Sp3 is a ubiquitous transcription factor closely related to Sp1 and contains activation and inhibitory domains. It can act as an activator or repressor of transcription [61,62]. In 2004, the results of Abdelrahim M et al. showed that Sp3 plays an important role in cell cycle progression of pancreatic cancer cells [63]. STAT5A is a transcription factor that mediates cytokine and hormone signals. Its constitutive activation has been observed in several human cancers, and it is oncogenic in cell culture models and transgenic animals [64]. Here, we identified the ability of c-Myc to act as an indirect positive regulator of SP3 and STAT5A expression. SP3 and STAT5A again mediate the indirect effects of c-Myc (Figure 6).

**Figure 7:**
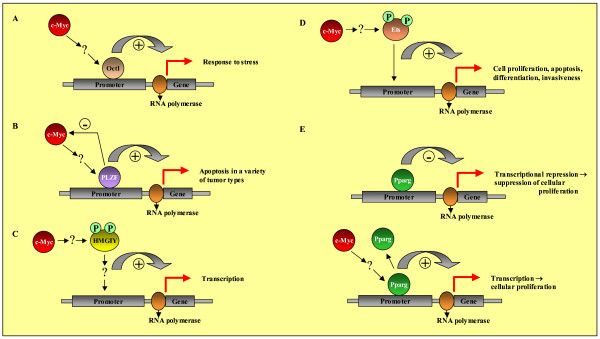
Putative indirect c-Myc actions, mediated by transcription factors regulated by overexpression of c-Myc on the protein level or mediated by as yet unknown mechanisms.

General analysis of the promoters which do not contain any putative c-Myc binding site nor any putative binding site of transcription factors (TFs) being transcriptionally induced by overexpression of c-Myc resulted in the observation that some TF binding sites are overrepresented against the control promoter background. These are binding sites of the TFs: OCT1, MZF1, PPARg, PLZF, ETS, and HMGIY (Figure 7).

Some of them are worth mentioning, because they seem in part to mediate the carcinogenic effect seen in mice after overexpression of the c-Myc oncogene: Oct1 modulates the activity of genes important for the cellular response to stress [65]. Although adipose tissue has been recognized as a principal site of PPAR gamma expression, it is now known that PPAR gamma is expressed in many other types of tissues and cells. It has often been mentioned in the context of cancer: its ligand activation has been shown to be involved in promotion or regression of colon tumors [66,67]. Furthermore, activation of PPAR gamma agonists capable of modestly inducing apoptosis has also been documented in a variety of tumor types [68]. Notably, Yamakawa-Karakida N et al. (2002) provided the first evidence of the linkage between PPAR gamma-mediated apoptosis and downregulation of c-Myc gene expression [69].

PLZF is known to be a transcriptional repressor which is associated with suppression of cellular proliferation. McConnell MJ et al. (2003) showed that PLZF expression maintains a cell in a quiescent state by repressing c-Myc expression and preventing cell cycle progression [70]. They suggested that loss of this suppression would have serious consequences for cell growth control and that growth suppression mediated by PLZF can be reversed by enforced expression of c-Myc. Here, through the overexpression of c-Myc, we found 37 putative target genes for PLZF. They are, however, transcriptionally induced, which might be the reversed effect mentioned by McConnell MJ et al. [70]. Under normal conditions, these genes would be transcriptionally repressed by PLZF. Loss of this repression might play a role in the development of the tumorigenic phenotype of c-Myc.

HMGIY has been shown to be a direct c-Myc target gene [71]. Some studies indicate an important role for HMGIY proteins in regulating gene expression [72]. Histon H1-mediated repression of transcription is reduced by HMGIY [73]. Like c-Myc, expression of HMGIY also correlates with rapidly proliferating mammalian tissues as well as neoplastic transformation [74] and, moreover, a higher residence time in heterochromatin and chromosomes, compared with euchromatic regions, correlates with an increased phosphorylation level of HMGIY [75].

The human Ets gene family includes 25 genes that code for transcription factors involved in the control of various aspects of cell proliferation, differentiation, and development. Ets domain transcription factors have been implicated in development of various forms of leukemias and solid tumors. It has been well established that their function can be controlled by phosphorylation-mediated effects on DNA binding. Phosphorylation has been shown to positively regulate transcriptional activities of Ets1 and Ets2. [76,77].

Binding sites of the transcription factors OCT1, MZF1, PPARg, PLZF, ETS, and HMGIY were found to be overrepresented in promoters of genes induced by overexpression of c-Myc. Their own gene expression, however, was unchanged. One explanation for this observation might be their regulation on the protein level. Nevertheless, some of these transcription factors seem to participate also in the Myc tumorigenic phenotype.

## Conclusion

Taken collectively, after transcription profiling of lung adenocarcinomas of female c-Myc-transgenic mice we were able to describe the c-Myc regulatory gene network in silico. By using positional weight matrices (PWMs), which is the most widely used method for recognition of transcription factor binding sites, we identified different mechanisms by which c-Myc putatively mediates its tumorigenic actions (see Figure 2):

1) Putative direct actions in part in cooperation or competition with other transcription factors (Figure 5).

2) Putative indirect c-Myc actions, mediated by transcription factors transcriptionally induced by overexpression of c-Myc (Figure 6).

3) Putative indirect c-Myc actions, mediated by transcription factors regulated by overexpression of c-Myc on the protein level or mediated by as yet unknown mechanisms (Figure 7).

Thus, our in silico description of the c-Myc regulatory gene network has yielded already known and also many novel putative direct and indirect targets of c-Myc. It provides some insights into how tumorigenesis is caused by deregulated c-Myc, a prevalent finding in human cancers.

## Methods

### Tissue samples

c-Myc-transgenic female mice displayed morphological alterations with varying degree of nuclear atypia, such as bronchiolo-adenomas and bronchiolo-adenocarcinomas. Thus, different stages of malignant transformation of alveolar epithelium were observed. In the non-transgenic control animals no abnormalities in lung tissue was detected with the exception of a single animal which showed a slight focal interstitial mononuclear cell infiltration.

### Gene expression studies

For gene expression analysis, either c-Myc-transgenics or non-transgenic controls were pooled such that 4 pools of 4 mice per group could be analyzed. Each pool was analysed in one microarray experiment. Only aliquots of individual RNA isolations were pooled, thus allowing measurement of selected genes by quantitative RT-PCR amongst all individual animals. Therefore, RNA was isolated from lung tissue of each individual animal, and identical amounts of RNA from 4 individuals of one group were pooled.

Transcriptome analysis was done according to the manufacturer's recommendation (Affymetrix GeneChip^® ^Expression Analysis Technical Manual (Santa Clara, CA)), using the GeneChip^® ^Test Arrays and GeneChip^® ^Mouse Genome 430 2.0. The frozen lung tissues (10–15 mg) were disrupted and homogenized using a rotor-stator homogenizer, and total RNA was isolated from the tissues using the RNeasy total RNA isolation kit (QIAGEN). RNA of individual samples was pooled as described above, and a second cleanup of isolated RNA was done using the RNeasy Mini Kit (QIAGEN). RNA was checked for quantity, purity, and integrity of the 18S and 28S ribosomal bands by capillary electrophoresis using the NanoDrop ND-1000 and the Agilent 2100 Bioanalyzer.

8 μg of total RNA were used as starting material to prepare cDNA. Synthesis of double-stranded cDNA was done with the GeneChip^® ^one-cycle cDNA Kit (Affymetrix). The cleanup of double-stranded cDNA was done using the GeneChip^® ^Sample Cleanup module (Affymetrix).

12 μl of cDNA solution were used for *in vitro *transcription. The *in vitro *transcription was conducted with the GeneChip^® ^IVT Labeling Kit (Affymetrix). The total amount of the reaction product was purified with the GeneChip^® ^Sample Cleanup module (Affymetrix). Purified cRNA was quantified and checked for quality using the NanoDrop ND-1000 and the Agilent 2100 Bioanalyzer. Purified cRNA was cleaved into fragments of 35–200 bases by metal-induced hydrolysis. The degree of fragmentation and the length distribution of the fragmented biotinylated cRNA were checked by capillary electrophoresis using the Agilent 2100 Bioanalyzer.

10 μg of biotinylated fragmented cRNA were hybridized onto the GeneChip^® ^Mouse Genome 430 2.0 array according to the manufacturer's recommendation. The hybridization was performed for 16 hours at 60 rpm and 45°C in the GeneChip^® ^Hybridization Oven 640 (Affymetrix). Washing and staining of the arrays was done on the GeneChip^® ^Fluidics Station 400 (Affymetrix) according to the manufacturer's recommendation. The antibody signal amplification, washing, and staining protocol (Affymetrix) was used to stain the arrays with streptavidin R-phycoerythrin (SAPE; Invitrogen, USA). To amplify staining, SAPE solution was added twice with a biotinylated anti-streptavidin antibody (Vector Laboratories, CA) staining step in between.

The arrays were scanned using the GeneChip^® ^Scanner 3000. Scanned image files were visually inspected for artifacts and then analyzed, each image being scaled to the same target value for comparison between chips. The GeneChip^® ^Operating Software (GCOS) was used to control the fluidics station and the scanner, to capture probe array data, and to analyze hybridization intensity data. Default parameters provided in the Affymetrix data analysis software package were applied for analysis.

After scanning, the GeneChip^® ^Operating Software (GCOS; version 1.1) generated the expression data for every single chip.

As detailed by the manufacturer, expression of a gene is corroborated by a set of 11 pairs of 25-oligomer. Next to perfect sequence matches, deliberate mismatches which differ by one base only in the middle of the oligomer are introduced to confirm hybridization products. A statistical expression algorithm within the GeneChip^® ^Operating Software (GCOS) calls on multiple perfect sequence matches and mismatches to determine the presence [a detection call "present" (P) or "absent" (A)] and abundance (a signal value) of an individual transcript. The detection (absolute information) and the signal (numerical values) are calculated independently.

To determine whether a gene is "significantly present", the average signal value (Signal-Avg) and the standard deviation [Stdev and Stdev(%)] were calculated using Affymetrix^® ^Data Mining Tool Software (version 3.1) and Microsoft^® ^Excel 2003. Additionally, the number of "present" calls (P-count) for each gene in four replicates was determined.

Criteria applied for a "significantly present" gene were, for example: average signal value ≥ 100, and all four "detection calls" must be "present" (P-count).

Multiple data from replicates were evaluated and compared using statistical analysis with the Affymetrix^® ^GeneChip^® ^Operating Software (GCOS) and Data Mining Tool (DMT). The average and standard deviation statistics within Affymetrix^® ^DMT were used to summarize the expression level (the signal values) for each transcript across the replicates. The unpaired t-test and comparison ranking were used to determine the direction and significance of change in a transcript's expression level between sets of replicates. Fold change values were calculated as the ratio of the average expression levels for each gene between c-Myc-transgenic animals and the correlating control experiment.

To extract genes with significantly altered expression, a comparison between groups of animals was conducted using the GeneChip^® ^Operating Software (GCOS). A comparison analysis was conducted for the female group within the c-Myc-transgenic line: transgenic versus non-transgenic strains.

For the comparison analysis, it was ensured that the scale factors for the compared chips did not differ by a factor larger than 3. The result of a single analysis between two different arrays was reported for each gene as "increase" (I) or "decrease" (D), and the change in signal intensity was determined as the signal logarithm ratio (log_2_ratio).

In this study, with four replicates per group, 16 comparison analyses (4 transgenic versus 4 non-transgenic) were conducted. Comparison ranking analysis was additionally done to study concordance between "increase calls" (I) or "decrease calls" (D) for replicates (this is counting the number of "I-calls" and "D-calls" out of 16).

The unpaired one-sided t-test converting the p-value to a two-sided p-value was used to determine the direction and significance of change in a transcript's expression level (Data Mining Tool, version 3.1). Signal values of each group were used as basis for calculation, with the original p-value cutoff determined to be 0.05.

Comparing different groups, a "fold change" (FC) was calculated, which is the ratio between the average signal values of groups to be compared. Ratios ≤ 1 were recalculated to give negative numbers whose magnitude resembles the extent of repression (for example: ratio of 0.5 was changed to -2).

To select genes in the c-Myc experiments, the following criteria were applied for the comparison conducted:

#### 1. For induced genes

○ the average signal value of the "treatment" had to be higher than 100

○ 4 P-calls had to be in the 4 "treatment" arrays

○ the fold change had to be 2.0 or higher (ratio of average signal values)

○ the result of the t-test had to be an "Up"-change call (p-value < 0.05) (based on single signal values of 4 replicates)

○ there had to be more than 13 (out of 16) induced calls in the comparison ranking

#### 2. For repressed genes

○ the average signal value of the "control" had to be higher than 100

○ 4 P-calls had to be in the 4 "control" arrays

○ the fold change had to be -2.0 or less (ratio of average signal values)

○ the result of the t-test had to be a "Down"-change call (p-value < 0.05) (based on single signal values of 4 replicates)

○ there had to be more than 13 (out of 16) decrease calls in the comparison ranking

Applying these criteria as detailed above, probe sets significantly altered in expression were selected. In a few cases, two or more of these "probe sets" were targeting the same gene.

To prevent reiterations, the following criteria were applied and only one "probe set" per gene was selected:

1. Primarily, "probe sets" not specific for one transcript were eliminated (indicated in the Probe Set ID by an additional letter, e.g. 1370470_**x**_at).

2. In case all probe sets were specific (Probe Set IDs without an additional letter, i.e. 138520_at) or all were not specific, those with higher signal values were selected.

### Real-time PCR

Real-time PCR measurement was done with the LightCycler^® ^(Roche Diagnostics, Penzberg, Germany). RNA was treated with Dnase and purified with RNeasy Mini Kit. Quality of purified RNA was analyzed in a denaturating Agarose gel. Reverse transcription (RT) was performed with 2 μg of RNA using Omniscript (Qiagen), RNase inhibitor and hexamers (Promega) in a final volume of 20 μl. RT reactions were diluted 1:4 and 2 μl was used for Real-time PCR. SYBR^® ^Green I was used as a fluorescent dye to determine the amplified PCR products after each cycle. The lengths of PCR products were checked in gel electrophoresis. PCR primers were synthesized by Invitrogen (Karlsruhe, Germany). At the end of each extension phase fluorescence was observed and used for quantitative measurements within the linear range of amplification yielding calculated concentrations as relative units. Exact quantification was achieved by serial dilution with cDNA produced from total RNA extracts using 1:5 or 1:3 dilution steps, depending on the expression level of the gene. Six runs were necessary to measure expression of the genes in all samples. For comparability of the six independent runs, standards were used, which were identical sample pools for all six runs. The standardized sample values for each gene of interest were divided through the standardized values of the housekeeping gene. As housekeeping gene, Ppib (peptidylprolyl isomerase B; cyclophilin B) was used.

### Sequence retrieval

The UCSC Genome Browser [78] was used to extract the promoter regions of regulated genes and promoter regions of control genes with no change in expression. Exclusively promoters of genes which are RefSeq annotated were extracted. The beginning of the first exon which also comprises the 5'UTR was considered to be a tentative TSS (transcription start site) [79]. 1000 bp upstream and 100 bp downstream of TSS were extracted, respectively. The choice of these regions was based on previous observations that c-Myc frequently binds to the regions having a distance of up to 1000 bps from the TSS [80,81]. It must be mentioned, however, that binding of c-Myc has also been proved to occur in the first intron of c-Myc target genes [82].

### Process of promoter analysis

The most widely used method for recognition of transcription factor binding sites is the application of positional weight matrices (PWMs) [34] TRANSFAC^® ^Professional rel. 10.1 is the largest collection of weight matrices for eukaryotic transcription factors [83,84] (BIOBASE GmbH, Wolfenbüttel, Germany). Here, the TRANSFAC^®^-integrated MATCH™ algorithm was employed, calculating scores for the matches by applying the so-called information vector [85]. The matrix profile "vertebrates_minSUM_highQual" was used. Default cutoff values for matrix similarity were used, whereas the cutoff values for core similarity were always set to 0.75. The matrix similarity cutoff is a score that describes the quality of a match between a matrix and an arbitrary part of the input sequences. In addition, only those matches which score higher than or equal to the matrix similarity threshold appear in the output. The number of transcription factor binding sites identified in the analyzed promoter set was compared to the number of transcription factor binding sites identified in a control promoter set [promoters of 100 selected genes which were not regulated at all in all four different groups]. The list of non-regulated genes was prepared after applying criteria described below and was included in Additional file 2.

### Selection of genes suitable as control promoters

For analysis of promoters of significantly altered genes, promoters of genes with no change in expression were selected. To do so, genes needed to be expressed with a signal value above 100, and the detection call of all 4 replicates had to be present. At the same time, the fold change must not be greater than 1.1 nor less than -1.1, the change direction, which is the result of the t-test with a p-value greater than 0.5, had to have a "None" call, and of the 16 comparison analyses conducted, less than five were allowed to have an "Induction" or a "Down" call. These criteria, applied to each comparison separately, in each case had to be true for all comparisons at the same time. They are summarized as follows:

○ the average signal value of the "treatment" had to be higher than 100

○ 4 P-calls had to be in the 4 "treatment" arrays

○ the fold change had to be between the range 1.1 and -1.1 (ratio of average signal values)

○ the result of the t-test had to be a "None"-change call

○ there had to be less than 5 (out of 16) induced calls in the comparison ranking

Applying these criteria for c-Myc as detailed above, 164 probe sets for genes with almost no change in expression were selected. In addition, 100 genes with a Transcript RefSeq number were selected to be used to extract promoter sequences for controls (Additional file 2).

## Authors' contributions

SR was responsible for the bioinformatical analysis of the study. JB initiated the study, and was responsible for the experimental part. Both authors drafted the manuscript, read and approved the final manuscript.

## Supplementary Material

Additional file 1 **Transcription profiling of lung adenocarcinomas of female c-Myc-transgenic mice: 477 significantly regulated genes**. This table shows the ProbeSet IDs, RefSeq accession numbers, Unigene IDs, gene titles, gene symbols, fold changes, and t-test p-values of the significantly regulated genes. Click here for file

Additional file 2 RefSeq IDs of genes the promoters of which were used as control promoters in promoter analysis. Click here for file

Additional file 3 **Putative direct c-Myc targets: positions of c-Myc binding sites in the promoter**. RefSeq accession numbers, gene symbols, and gene titles of the putative c-Myc targets are listed in this table. Moreover, the TRANSFAC identifier of the respective matrix, the position of the hit (1 = 1000 bp upstream of TSS; 1100 = 100 bp downstream of TSS), and the corresponding recognized sequence are included in the right column. Click here for file

Additional file 4 110 randomly extracted 205-bp sequences from the control promoters which were not regulated at all. Click here for file

Additional file 5 **Complete results: analysis of flanking sequences (+/- 100 bp) around c-Myc binding sites**. This table shows the TRANSFAC identifier of the applied matrices, the number of hits identified in the sequences of the induced gene promoters, the number of hits identified in the sequences of the control gene promoters, and the corresponding fold occurrences of hits. Click here for file

Additional file 6 36 deregulated genes possessing transcription factor or transcription regulator activity. Click here for file

Additional file 7 **Analysis of promoters of 361 induced genes with respect to binding sites of TFs which were transcriptionally induced by c-Myc overexpression**. RefSeq accession numbers, TRANSFAC identifier of the respective matrix, the position of the hit (1 = 1000 bp upstream of TSS; 1100 = 100 bp downstream of TSS) and the corresponding recognized sequence are given in this table. Click here for file

Additional file 8 **Putative target genes identified in silico by using positional weight matrices**. This table shows the RefSeq accession numbers, gene titles, and gene symbols of the putative target genes identified for the respective promoter analysis. Click here for file

Additional file 9 **Putative target genes of c-Myc and of transcription factors the expression of which was induced by c-Myc**. This table shows the RefSeq accession numbers of genes in the promoters of which the matrix hits were found, the TRANSFAC identifier of the respective matrices, and the location and corresponding sequence of the matrix hits. Click here for file

## References

[B1] Vogelstein B, Kinzler KW (2004). Cancer genes and the pathways they control. Nat Med.

[B2] Nesbit CE, Tersak JM, Prochownik EV (1999). MYC oncogenes and human neoplastic disease. Oncogene.

[B3] Henriksson M, Luscher B (1996). Proteins of the Myc network: essential regulators of cell growth and differentiation. Adv Cancer Res.

[B4] Pelengaris S, Khan M, Evan G (2002). c-MYC: more than just a matter of life and death. Nat Rev Cancer.

[B5] Moroy T, Verbeek S, Ma A, Achacoso P, Berns A, Alt F (1991). E mu N- and E mu L-myc cooperate with E mu pim-1 to generate lymphoid tumors at high frequency in double-transgenic mice. Oncogene.

[B6] Zhang X, Lee C, Ng PY, Rubin M, Shabsigh A, Buttyan R (2000). Prostatic neoplasia in transgenic mice with prostate-directed overexpression of the c-myc oncoprotein. Prostate.

[B7] Jensen NA, Pedersen KM, Lihme F, Rask L, Nielsen JV, Rasmussen TE, Mitchelmore C (2003). Astroglial c-Myc overexpression predisposes mice to primary malignant gliomas. J Biol Chem.

[B8] Blackwood EM, Eisenman RN (1991). Max: a helix-loop-helix zipper protein that forms a sequence-specific DNA-binding complex with Myc. Science.

[B9] Claassen GF, Hann SR (1999). Myc-mediated transformation: the repression connection. Oncogene.

[B10] Brenner C, Deplus R, Didelot C, Loriot A, Vire E, De Smet C, Gutierrez A, Danovi D, Bernard D, Boon T, Pelicci PG, Amati B, Kouzarides T, de Launoit Y, Di Croce L, Fuks F (2005). Myc represses transcription through recruitment of DNA methyltransferase corepressor. EMBO J.

[B11] Amati B, Littlewood TD, Evan GI, Land H (1993). The c-Myc protein induces cell cycle progression and apoptosis through dimerization with Max. EMBO J.

[B12] Bar-Ner M, Messing LT, Cultraro CM, Birrer MJ, Segal S (1992). Regions within the c-Myc protein that are necessary for transformation are also required for inhibition of differentiation of murine erythroleukemia cells. Cell Growth Differ.

[B13] Cole MD, McMahon SB (1999). The Myc oncoprotein: a critical evaluation of transactivation and target gene regulation. Oncogene.

[B14] Brough DE, Hofmann TJ, Ellwood KB, Townley RA, Cole MD (1995). An essential domain of the c-myc protein interacts with a nuclear factor that is also required for E1A-mediated transformation. Mol Cell Biol.

[B15] Bello-Fernandez C, Packham G, Cleveland JL (1993). The ornithine decarboxylase gene is a transcriptional target of c-Myc. Proc Natl Acad Sci USA.

[B16] Levens D (2002). Disentangling the MYC web. Proc Natl Acad Sci USA.

[B17] Boon K, Caron HN, van Asperen R, Valentijn L, Hermus MC, van Sluis P, Roobeek I, Weis I, Voute PA, Schwab M, Versteeg R (2001). N-myc enhances the expression of a large set of genes functioning in ribosome biogenesis and protein synthesis. EMBO J.

[B18] Coller HA, Grandori C, Tamayo P, Colbert T, Lander ES, Eisenman RN, Golub TR (2000). Expression analysis with oligonucleotide microarrays reveals that MYC regulates genes involved in growth, cell cycle, signaling, and adhesion. Proc Natl Acad Sci USA.

[B19] Guo QM, Malek RL, Kim S, Chiao C, He M, Ruffy M, Sanka K, Lee NH, Dang CV, Liu ET (2000). Identification of c-myc responsive genes using rat cDNA microarray. Cancer Res.

[B20] Menssen A, Hermeking H (2002). Characterization of the c-MYC-regulated transcriptome by SAGE: identification and analysis of c-MYC target genes. Proc Natl Acad Sci USA.

[B21] Nesbit CE, Tersak JM, Grove LE, Drzal A, Choi H, Prochownik EV (2000). Genetic dissection of c-myc apoptotic pathways. Oncogene.

[B22] Remondini D, O'Connell B, Intrator N, Sedivy JM, Neretti N, Castellani GC, Cooper LN (2005). Targeting c-Myc-activated genes with a correlation method: detection of global changes in large gene expression network dynamics. Proc Natl Acad Sci USA.

[B23] Schuhmacher M, Kohlhuber F, Holzel M, Kaiser C, Burtscher H, Jarsch M, Bornkamm GW, Laux G, Polack A, Weidle UH, Eick D (2001). The transcriptional program of a human B cell line in response to Myc. Nucleic Acids Res.

[B24] Schuldiner O, Benvenisty N (2001). A DNA microarray screen for genes involved in c-MYC and N-MYC oncogenesis in human tumors. Oncogene.

[B25] Watson JD, Oster SK, Shago M, Khosravi F, Penn LZ (2002). Identifying genes regulated in a Myc-dependent manner. J Biol Chem.

[B26] Myc Cancer Gene. http://www.myc-cancer-gene.org/.

[B27] Zeller KI, Jegga AG, Aronow BJ, O'Donnell KA, Dang CV (2003). An integrated database of genes responsive to the Myc oncogenic transcription factor: identification of direct genomic targets. Genome Biol.

[B28] Gaubatz S, Imhof A, Dosch R, Werner O, Mitchell P, Buettner R, Eilers M (1995). Transcriptional activation by Myc is under negative control by the transcription factor AP-2. EMBO J.

[B29] Zeller KI, Zhao X, Lee CW, Chiu KP, Yao F, Yustein JT, Ooi HS, Orlov YL, Shahab A, Yong HC, Fu Y, Weng Z, Kuznetsov VA, Sung WK, Ruan Y, Dang CV, Wei CL (2006). Global mapping of c-Myc binding sites and target gene networks in human B cells. Proc Natl Acad Sci USA.

[B30] Gene Expression Omnibus (GEO). http://www.myc-cancer-gene.org/.

[B31] Basso K, Margolin AA, Stolovitzky G, Klein U, Dalla-Favera R, Califano A (2005). Reverse engineering of regulatory networks in human B cells. Nat Genet.

[B32] Chen Y, Blackwell TW, Chen J, Gao J, Lee AW, States DJ (2007). Integration of genome and chromatin structure with gene expression profiles to predict c-MYC recognition site binding and function. PLoS Comput Biol.

[B33] Watson JD, Oster SK, Shago M, Khosravi F, Penn LZ (2002). Identifying genes regulated in a Myc-dependent manner. J Biol Chem.

[B34] Quandt K, Frech K, Karas H, Wingender E, Werner T (1995). MatInd and MatInspector: new fast and versatile tools for detection of consensus matches in nucleotide sequence data. Nucleic Acids Res.

[B35] Whitlock JP (1999). Induction of cytochrome P4501A1. Annu Rev Pharmacol Toxicol.

[B36] Elkon R, Zeller KI, Linhart C, Dang CV, Shamir R, Shiloh Y (2004). In silico identification of transcriptional regulators associated with c-Myc. Nucleic Acids Res.

[B37] Sears RC, Nevins JR (2002). Signaling networks that link cell proliferation and cell fate. J Biol Chem.

[B38] Hiebert SW, Lipp M, Nevins JR (1989). E1A-dependent trans-activation of the human MYC promoter is mediated by the E2F factor. Proc Natl Acad Sci USA.

[B39] Hsiao KM, McMahon SL, Farnham PJ (1994). Multiple DNA elements are required for the growth regulation of the mouse E2F1 promoter. Genes Dev.

[B40] Wu F, Lee AS (2001). YY1 as a regulator of replication-dependent hamster histone H3.2 promoter and an interactive partner of AP-2. J Biol Chem.

[B41] Decary S, Decesse JT, Ogryzko V, Reed JC, Naguibneva I, Harel-Bellan A, Cremisi CE (2002). The retinoblastoma protein binds the promoter of the survival gene bcl-2 and regulates its transcription in epithelial cells through transcription factor AP-2. Mol Cell Biol.

[B42] Numoto M, Niwa O, Kaplan J, Wong KK, Merrell K, Kamiya K, Yanagihara K, Calame K (1993). Transcriptional repressor ZF5 identifies a new conserved domain in zinc finger proteins. Nucleic Acids Res.

[B43] Kaplan J, Calame K (1997). The ZiN/POZ domain of ZF5 is required for both transcriptional activation and repression. Nucleic Acids Res.

[B44] Yokoro K, Yanagidani A, Obata T, Yamamoto S, Numoto M (1998). Genomic cloning and characterization of the mouse POZ/zinc-finger protein ZF5. Biochem Biophys Res Commun.

[B45] Imai KS, Satou Y, Satoh N (2002). Multiple functions of a Zic-like gene in the differentiation of notochord, central nervous system and muscle in Ciona savignyi embryos. Development.

[B46] Wagner EF (2001). AP-1 – Introductory remarks. Oncogene.

[B47] Gupta S, Campbell D, Derijard B, Davis RJ (1995). Transcription factor ATF2 regulation by the JNK signal transduction pathway. Science.

[B48] Hayakawa J, Mittal S, Wang Y, Korkmaz KS, Adamson E, English C, Ohmichi M, McClelland M, Mercola D (2004). Identification of promoters bound by c-Jun/ATF2 during rapid large-scale gene activation following genotoxic stress. Mol Cell.

[B49] Miethe J, Schwartz C, Wottrich K, Wenning D, Klempnauer KH (2001). Crosstalk between Myc and activating transcription factor 2 (ATF2): Myc prolongs the half-life and induces phosphorylation of ATF2. Oncogene.

[B50] Tamura K, Hua B, Adachi S, Guney I, Kawauchi J, Morioka M, Tamamori-Adachi M, Tanaka Y, Nakabeppu Y, Sunamori M, Sedivy JM, Kitajima S (2005). Stress response gene ATF3 is a target of c-myc in serum-induced cell proliferation. EMBO J.

[B51] Kaestner KH, Knochel W, Martinez DE (2000). Unified nomenclature for the winged helix/forkhead transcription factors. Genes Dev.

[B52] Mahlapuu M, Pelto-Huikko M, Aitola M, Enerback S, Carlsson P (1998). FREAC-1 contains a cell-type-specific transcriptional activation domain and is expressed in epithelial-mesenchymal interfaces. Dev Biol.

[B53] Kalinichenko VV, Lim L, Shin B, Costa RH (2001). Differential expression of forkhead box transcription factors following butylated hydroxytoluene lung injury. Am J Physiol Lung Cell Mol Physiol.

[B54] Frederick JP, Liberati NT, Waddell DS, Shi Y, Wang XF (2004). Transforming growth factor beta-mediated transcriptional repression of c-myc is dependent on direct binding of Smad3 to a novel repressive Smad binding element. Mol Cell Biol.

[B55] Lim SK, Hoffmann FM (2006). Smad4 cooperates with lymphoid enhancer-binding factor 1/T cell-specific factor to increase c-myc expression in the absence of TGF-beta signaling. Proc Natl Acad Sci USA.

[B56] Ahn SG, Cho GH, Jeong SY, Rhim H, Choi JY, Kim IK (1999). Identification of cDNAs for Sox-4, an HMG-Box protein, and a novel human homolog of yeast splicing factor SSF-1 differentially regulated during apoptosis induced by prostaglandin A2/delta12-PGJ2 in Hep3B cells. Biochem Biophys Res Commun.

[B57] Lee CJ, Appleby VJ, Orme AT, Chan WI, Scotting PJ (2002). Differential expression of SOX4 and SOX11 in medulloblastoma. J Neurooncol.

[B58] Frierson HF, El Naggar AK, Welsh JB, Sapinoso LM, Su AI, Cheng J, Saku T, Moskaluk CA, Hampton GM (2002). Large scale molecular analysis identifies genes with altered expression in salivary adenoid cystic carcinoma. Am J Pathol.

[B59] Lund AH, Turner G, Trubetskoy A, Verhoeven E, Wientjens E, Hulsman D, Russell R, DePinho RA, Lenz J, van Lohuizen M (2002). Genome-wide retroviral insertional tagging of genes involved in cancer in Cdkn2a-deficient mice. Nat Genet.

[B60] Suzuki T, Shen H, Akagi K, Morse HC, Malley JD, Naiman DQ, Jenkins NA, Copeland NG (2002). New genes involved in cancer identified by retroviral tagging. Nat Genet.

[B61] Braun H, Koop R, Ertmer A, Nacht S, Suske G (2001). Transcription factor Sp3 is regulated by acetylation. Nucleic Acids Res.

[B62] Birnbaum MJ, van Wijnen AJ, Odgren PR, Last TJ, Suske G, Stein GS, Stein JL (1995). Sp1 trans-activation of cell cycle regulated promoters is selectively repressed by Sp3. Biochemistry.

[B63] Abdelrahim M, Smith R, Burghardt R, Safe S (2004). Role of Sp proteins in regulation of vascular endothelial growth factor expression and proliferation of pancreatic cancer cells. Cancer Res.

[B64] Bowman T, Garcia R, Turkson J, Jove R (2000). STATs in oncogenesis. Oncogene.

[B65] Tantin D, Schild-Poulter C, Wang V, Hache RJ, Sharp PA (2005). The octamer binding transcription factor Oct-1 is a stress sensor. Cancer Res.

[B66] Gupta RA, Dubois RN (2002). Controversy: PPARgamma as a target for treatment of colorectal cancer. Am J Physiol Gastrointest Liver Physiol.

[B67] Lefebvre AM, Laville M, Vega N, Riou JP, van Gaal L, Auwerx J, Vidal H (1998). Depot-specific differences in adipose tissue gene expression in lean and obese subjects. Diabetes.

[B68] Sato H, Ishihara S, Kawashima K, Moriyama N, Suetsugu H, Kazumori H, Okuyama T, Rumi MA, Fukuda R, Nagasue N, Kinoshita Y (2000). Expression of peroxisome proliferator-activated receptor (PPAR)gamma in gastric cancer and inhibitory effects of PPARgamma agonists. Br J Cancer.

[B69] Yamakawa-Karakida N, Sugita K, Inukai T, Goi K, Nakamura M, Uno K, Sato H, Kagami K, Barker N, Nakazawa S (2002). Ligand activation of peroxisome proliferator-activated receptor gamma induces apoptosis of leukemia cells by down-regulating the c-myc gene expression via blockade of the Tcf-4 activity. Cell Death Differ.

[B70] McConnell MJ, Chevallier N, Berkofsky-Fessler W, Giltnane JM, Malani RB, Staudt LM, Licht JD (2003). Growth suppression by acute promyelocytic leukemia-associated protein PLZF is mediated by repression of c-myc expression. Mol Cell Biol.

[B71] Wood LJ, Mukherjee M, Dolde CE, Xu Y, Maher JF, Bunton TE, Williams JB, Resar LM (2000). HMG-I/Y, a new c-Myc target gene and potential oncogene. Mol Cell Biol.

[B72] Falvo JV, Thanos D, Maniatis T (1995). Reversal of intrinsic DNA bends in the IFN beta gene enhancer by transcription factors and the architectural protein HMG I(Y). Cell.

[B73] Zhao K, Kas E, Gonzalez E, Laemmli UK (1993). SAR-dependent mobilization of histone H1 by HMG-I/Y in vitro: HMG-I/Y is enriched in H1-depleted chromatin. EMBO J.

[B74] Tamimi Y, Poel HG van der, Karthaus HF, Debruyne FM, Schalken JA (1996). A retrospective study of high mobility group protein I(Y) as progression marker for prostate cancer determined by in situ hybridization. Br J Cancer.

[B75] Harrer M, Luhrs H, Bustin M, Scheer U, Hock R (2004). Dynamic interaction of HMGA1a proteins with chromatin. Journal of Cell Science.

[B76] Hsu T, Trojanowska M, Watson DK (2004). Ets proteins in biological control and cancer. J Cell Biochem.

[B77] Oikawa T, Yamada T (2003). Molecular biology of the Ets family of transcription factors. Gene.

[B78] UCSC Genome Bioinformatics. http://genome.ucsc.edu/.

[B79] Coleman SL, Buckland PR, Hoogendoorn B, Guy C, Smith K, O'Donovan MC (2002). Experimental analysis of the annotation of promoters in the public database. Hum Mol Genet.

[B80] Li Z, Van Calcar S, Qu C, Cavenee WK, Zhang MQ, Ren B (2003). A global transcriptional regulatory role for c-Myc in Burkitt's lymphoma cells. Proc Natl Acad Sci USA.

[B81] Parisi F, Wirapati P, Naef F (2007). Identifying synergistic regulation involving c-Myc and sp1 in human tissues. Nucleic Acids Res.

[B82] Haggerty TJ, Zeller KI, Osthus RC, Wonsey DR, Dang CV (2003). A strategy for identifying transcription factor binding sites reveals two classes of genomic c-Myc target sites. Proc Natl Acad Sci USA.

[B83] BIOBASE Biological Databases. http://www.biobase-international.com/pages/.

[B84] Wingender E, Chen X, Fricke E, Geffers R, Hehl R, Liebich I, Krull M, Matys V, Michael H, Ohnhauser R, Pruss M, Schacherer F, Thiele S, Urbach S (2001). The TRANSFAC system on gene expression regulation. Nucleic Acids Res.

[B85] Kel AE, Gossling E, Reuter I, Cheremushkin E, Kel-Margoulis OV, Wingender E (2003). MATCH: A tool for searching transcription factor binding sites in DNA sequences. Nucleic Acids Res.

