# Supplementary material for: Transcription profiling of lung adenocarcinomas of c-myc-transgenic mice: Identification of the c-myc regulatory gene network
Source: BMC Syst Biol. 2008 May 22;2:46. doi: 10.1186/1752-0509-2-46 (PMC2430022; doi:10.1186/1752-0509-2-46)
Supplement: Additional file 5 — Complete results: analysis of flanking sequences (+/- 100 bp) around c-Myc binding sites. This table shows the TRANSFAC identifier of the applied matrices, the number of hits identified in the sequences of the induced gene promoters, the number of hits identified in the sequences of the control gene promoters, and the corresponding fold occurrences of hits. [file 1752-0509-2-46-S5.doc]

| **Matrix** | **Number of hits in induced promoters** | **Number of hits in control promoters** | **Fold-Occurrence** |
| --- | --- | --- | --- |
| V$USF_Q6 | 68 | 2 | 34.00 |
| V$ARNT_01 | 28 | 1 | 28.00 |
| V$USF_01 | 82 | 4 | 20.50 |
| V$USF_Q6_01 | 39 | 2 | 19.50 |
| V$ARNT_02 | 168 | 10 | 16.80 |
| V$USF2_Q6 | 168 | 10 | 16.80 |
| V$MYC_Q2 | 96 | 7 | 13.71 |
| V$USF_C | 93 | 7 | 13.29 |
| V$EBOX_Q6_01 | 25 | 2 | 12.50 |
| V$USF_02 | 86 | 8 | 10.75 |
| V$AP2GAMMA_01 | 21 | 2 | 10.50 |
| V$MYCMAX_02 | 10 | 1 | 10.00 |
| V$MYCMAX_B | 18 | 2 | 9.00 |
| V$E2F1DP1RB_01 | 6 | 1 | 6.00 |
| V$MAX_01 | 34 | 6 | 5.67 |
| V$AHR_01 | 4 | 1 | 4.00 |
| V$MYCMAX_01 | 16 | 4 | 4.00 |
| V$WT1_Q6 | 12 | 3 | 4.00 |
| V$XFD1_01 | 4 | 1 | 4.00 |
| V$AP2ALPHA_03 | 30 | 8 | 3.75 |
| V$E2F1_Q3_01 | 11 | 3 | 3.67 |
| V$MAZ_Q6 | 14 | 4 | 3.50 |
| V$TCF4_Q5 | 7 | 2 | 3.50 |
| V$E2F1_Q3 | 22 | 7 | 3.14 |
| V$AHRARNT_02 | 6 | 2 | 3.00 |
| V$GATA4_Q3 | 6 | 2 | 3.00 |
| V$HMEF2_Q6 | 3 | 1 | 3.00 |
| V$IK2_01 | 6 | 2 | 3.00 |
| V$SREBP1_02 | 12 | 4 | 3.00 |
| V$AP2_Q6_01 | 14 | 5 | 2.80 |
| V$MYOGENIN_Q6 | 5 | 2 | 2.50 |
| V$YY1_Q6_02 | 5 | 2 | 2.50 |
| V$AP2_Q6 | 17 | 7 | 2.43 |
| V$MUSCLE_INI_B | 12 | 5 | 2.40 |
| V$E2F_Q2 | 50 | 21 | 2.38 |
| V$AP2REP_01 | 7 | 3 | 2.33 |
| V$ZF5_B | 85 | 37 | 2.30 |
| V$E2F1_Q4 | 9 | 4 | 2.25 |
| V$E2F1_Q6 | 11 | 5 | 2.20 |
| V$ZIC3_01 | 22 | 10 | 2.20 |
| V$ATF1_Q6 | 2 | 1 | 2.00 |
| V$GATA2_02 | 4 | 2 | 2.00 |
| V$HEB_Q6 | 2 | 1 | 2.00 |
| V$HNF4_01_B | 2 | 1 | 2.00 |
| V$MINI20_B | 6 | 3 | 2.00 |
| V$NFAT_Q6 | 2 | 1 | 2.00 |
| V$NFY_01 | 6 | 3 | 2.00 |
| V$OCT4_01 | 2 | 1 | 2.00 |
| V$SMAD_Q6_01 | 2 | 1 | 2.00 |
| V$SREBP1_Q6 | 8 | 4 | 2.00 |
| V$ZF5_01 | 66 | 34 | 1.94 |
| V$VDR_Q3 | 13 | 7 | 1.86 |
| V$GC_01 | 29 | 16 | 1.81 |
| V$PAX5_02 | 27 | 15 | 1.80 |
| V$AP2_Q3 | 14 | 8 | 1.75 |
| V$EGR2_01 | 7 | 4 | 1.75 |
| V$KROX_Q6 | 7 | 4 | 1.75 |
| V$TFIII_Q6 | 7 | 4 | 1.75 |
| V$ETS_Q4 | 5 | 3 | 1.67 |
| V$MAZR_01 | 5 | 3 | 1.67 |
| V$TCF11MAFG_01 | 10 | 6 | 1.67 |
| V$YY1_Q6 | 5 | 3 | 1.67 |
| V$CP2_01 | 13 | 8 | 1.63 |
| V$PAX2_01 | 8 | 5 | 1.60 |
| V$CHCH_01 | 33 | 21 | 1.57 |
| V$COUP_01 | 3 | 2 | 1.50 |
| V$ETS1_B | 9 | 6 | 1.50 |
| V$FREAC2_01 | 3 | 2 | 1.50 |
| V$GATA1_06 | 3 | 2 | 1.50 |
| V$HNF3B_01 | 3 | 2 | 1.50 |
| V$HNF4ALPHA_Q6 | 6 | 4 | 1.50 |
| V$MEF2_01 | 3 | 2 | 1.50 |
| V$MINI19_B | 3 | 2 | 1.50 |
| V$MZF1_02 | 3 | 2 | 1.50 |
| V$PIT1_Q6 | 3 | 2 | 1.50 |
| V$PPARA_02 | 3 | 2 | 1.50 |
| V$SPZ1_01 | 9 | 6 | 1.50 |
| V$SZF11_01 | 9 | 6 | 1.50 |
| V$XFD3_01 | 3 | 2 | 1.50 |
| V$SP1_Q4_01 | 20 | 14 | 1.43 |
| V$EGR1_01 | 7 | 5 | 1.40 |
| V$PLZF_02 | 7 | 5 | 1.40 |
| V$PPARA_01 | 7 | 5 | 1.40 |
| V$SMAD4_Q6 | 7 | 5 | 1.40 |
| V$MYOGNF1_01 | 25 | 18 | 1.39 |
| V$ZIC1_01 | 11 | 8 | 1.38 |
| V$CACD_01 | 15 | 11 | 1.36 |
| V$ETS_Q6 | 4 | 3 | 1.33 |
| V$HELIOSA_02 | 4 | 3 | 1.33 |
| V$OCT1_02 | 4 | 3 | 1.33 |
| V$TBX5_01 | 4 | 3 | 1.33 |
| V$SP1_Q6 | 18 | 14 | 1.29 |
| V$SP1_Q6_01 | 18 | 14 | 1.29 |
| V$SP1_Q2_01 | 14 | 11 | 1.27 |
| V$SP1_01 | 19 | 15 | 1.27 |
| V$ETF_Q6 | 21 | 17 | 1.24 |
| V$GABP_B | 17 | 14 | 1.21 |
| V$BRCA_01 | 6 | 5 | 1.20 |
| V$CMYB_01 | 6 | 5 | 1.20 |
| V$SMAD_Q6 | 6 | 5 | 1.20 |
| V$FAC1_01 | 7 | 6 | 1.17 |
| V$NFY_C | 7 | 6 | 1.17 |
| V$SOX10_Q6 | 7 | 6 | 1.17 |
| V$CETS1P54_03 | 22 | 19 | 1.16 |
| V$TAXCREB_02 | 8 | 7 | 1.14 |
| V$CETS168_Q6 | 10 | 9 | 1.11 |
| V$CETS1P54_01 | 10 | 9 | 1.11 |
| V$PAX5_01 | 10 | 9 | 1.11 |
| V$AML1_01 | 14 | 13 | 1.08 |
| V$AML1_Q6 | 14 | 13 | 1.08 |
| V$PAX6_01 | 18 | 17 | 1.06 |
| V$AHRHIF_Q6 | 3 | 3 | 1.00 |
| V$AMEF2_Q6 | 1 | 1 | 1.00 |
| V$AP1_Q4_01 | 1 | 1 | 1.00 |
| V$AP2ALPHA_02 | 5 | 5 | 1.00 |
| V$AP4_Q6_01 | 2 | 2 | 1.00 |
| V$AR_01 | 2 | 2 | 1.00 |
| V$AREB6_01 | 4 | 4 | 1.00 |
| V$ARP1_01 | 1 | 1 | 1.00 |
| V$ATF_B | 2 | 2 | 1.00 |
| V$ATF3_Q6 | 1 | 1 | 1.00 |
| V$CBF_01 | 2 | 2 | 1.00 |
| V$CBF_02 | 2 | 2 | 1.00 |
| V$DEAF1_01 | 2 | 2 | 1.00 |
| V$DR4_Q2 | 1 | 1 | 1.00 |
| V$E47_01 | 10 | 10 | 1.00 |
| V$ELK1_01 | 4 | 4 | 1.00 |
| V$FOXO1_02 | 1 | 1 | 1.00 |
| V$GATA1_02 | 1 | 1 | 1.00 |
| V$GRE_C | 4 | 4 | 1.00 |
| V$HES1_Q2 | 1 | 1 | 1.00 |
| V$HFH8_01 | 2 | 2 | 1.00 |
| V$HIF1_Q5 | 1 | 1 | 1.00 |
| V$HNF3ALPHA_Q6 | 4 | 4 | 1.00 |
| V$HNF4_DR1_Q3 | 2 | 2 | 1.00 |
| V$ICSBP_Q6 | 1 | 1 | 1.00 |
| V$ISRE_01 | 1 | 1 | 1.00 |
| V$LBP1_Q6 | 4 | 4 | 1.00 |
| V$LDSPOLYA_B | 1 | 1 | 1.00 |
| V$LEF1_Q2 | 16 | 16 | 1.00 |
| V$MSX1_01 | 9 | 9 | 1.00 |
| V$NFKAPPAB50_01 | 2 | 2 | 1.00 |
| V$NFY_Q6_01 | 4 | 4 | 1.00 |
| V$NKX25_Q5 | 2 | 2 | 1.00 |
| V$OCT_Q6 | 2 | 2 | 1.00 |
| V$OCT1_04 | 1 | 1 | 1.00 |
| V$OCT1_Q5_01 | 1 | 1 | 1.00 |
| V$P53_01 | 1 | 1 | 1.00 |
| V$PBX1_03 | 3 | 3 | 1.00 |
| V$POU3F2_02 | 4 | 4 | 1.00 |
| V$PPARG_03 | 1 | 1 | 1.00 |
| V$RFX_Q6 | 5 | 5 | 1.00 |
| V$RFX1_02 | 7 | 7 | 1.00 |
| V$SOX9_B1 | 1 | 1 | 1.00 |
| V$SP3_Q3 | 3 | 3 | 1.00 |
| V$STAF_01 | 1 | 1 | 1.00 |
| V$TFIIA_Q6 | 1 | 1 | 1.00 |
| V$XFD2_01 | 1 | 1 | 1.00 |
| V$YY1_02 | 4 | 4 | 1.00 |
| V$HAND1E47_01 | 12 | 13 | 0.92 |
| V$CP2_02 | 11 | 12 | 0.92 |
| V$VMAF_01 | 11 | 12 | 0.92 |
| V$ELK1_02 | 16 | 18 | 0.89 |
| V$PEA3_Q6 | 7 | 8 | 0.88 |
| V$MOVOB_01 | 12 | 14 | 0.86 |
| V$PU1_Q6 | 6 | 7 | 0.86 |
| V$E2F_01 | 5 | 6 | 0.83 |
| V$LRF_Q2 | 5 | 6 | 0.83 |
| V$MTATA_B | 5 | 6 | 0.83 |
| V$TTF1_Q6 | 5 | 6 | 0.83 |
| V$IK3_01 | 14 | 17 | 0.82 |
| V$ALPHACP1_01 | 4 | 5 | 0.80 |
| V$CAAT_01 | 4 | 5 | 0.80 |
| V$FOXO4_02 | 4 | 5 | 0.80 |
| V$VMYB_01 | 8 | 10 | 0.80 |
| V$ATF_01 | 3 | 4 | 0.75 |
| V$CEBP_Q2_01 | 3 | 4 | 0.75 |
| V$E2A_Q6 | 3 | 4 | 0.75 |
| V$OCT1_03 | 3 | 4 | 0.75 |
| V$ETS2_B | 8 | 11 | 0.73 |
| V$TEF1_Q6 | 7 | 10 | 0.70 |
| V$AP1_Q4 | 2 | 3 | 0.67 |
| V$CDPCR3HD_01 | 2 | 3 | 0.67 |
| V$CEBPA_01 | 2 | 3 | 0.67 |
| V$FREAC7_01 | 2 | 3 | 0.67 |
| V$GATA2_03 | 2 | 3 | 0.67 |
| V$HNF3_Q6_01 | 4 | 6 | 0.67 |
| V$IRF1_Q6 | 2 | 3 | 0.67 |
| V$MMEF2_Q6 | 2 | 3 | 0.67 |
| V$NFY_Q6 | 2 | 3 | 0.67 |
| V$STAT5A_02 | 4 | 6 | 0.67 |
| V$TBP_Q6 | 2 | 3 | 0.67 |
| V$TITF1_Q3 | 4 | 6 | 0.67 |
| V$PPARG_02 | 7 | 11 | 0.64 |
| V$AIRE_01 | 6 | 10 | 0.60 |
| V$FOXO3_01 | 3 | 5 | 0.60 |
| V$RFX1_01 | 3 | 5 | 0.60 |
| V$CDX2_Q5 | 8 | 14 | 0.57 |
| V$NERF_Q2 | 5 | 9 | 0.56 |
| V$GR_Q6 | 9 | 17 | 0.53 |
| V$CART1_01 | 1 | 2 | 0.50 |
| V$CEBP_C | 2 | 4 | 0.50 |
| V$CEBPB_02 | 1 | 2 | 0.50 |
| V$CEBPGAMMA_Q6 | 1 | 2 | 0.50 |
| V$CREB_Q2 | 1 | 2 | 0.50 |
| V$E2F1_Q6_01 | 1 | 2 | 0.50 |
| V$EFC_Q6 | 2 | 4 | 0.50 |
| V$ER_Q6 | 1 | 2 | 0.50 |
| V$FREAC4_01 | 1 | 2 | 0.50 |
| V$GATA1_05 | 1 | 2 | 0.50 |
| V$HNF3_Q6 | 3 | 6 | 0.50 |
| V$IK1_01 | 1 | 2 | 0.50 |
| V$IRF7_01 | 1 | 2 | 0.50 |
| V$MEIS1AHOXA9_01 | 3 | 6 | 0.50 |
| V$NRF2_Q4 | 1 | 2 | 0.50 |
| V$OCT_C | 2 | 4 | 0.50 |
| V$PAX6_Q2 | 1 | 2 | 0.50 |
| V$PBX_Q3 | 1 | 2 | 0.50 |
| V$SREBP_Q3 | 2 | 4 | 0.50 |
| V$TAL1ALPHAE47_01 | 2 | 4 | 0.50 |
| V$TBP_01 | 1 | 2 | 0.50 |
| V$BRN2_01 | 3 | 7 | 0.43 |
| V$TATA_01 | 3 | 7 | 0.43 |
| V$AP1_C | 4 | 10 | 0.40 |
| V$CREBP1CJUN_01 | 2 | 5 | 0.40 |
| V$DBP_Q6 | 2 | 5 | 0.40 |
| V$DR3_Q4 | 2 | 5 | 0.40 |
| V$MEIS1BHOXA9_02 | 2 | 5 | 0.40 |
| V$PAX8_01 | 7 | 18 | 0.39 |
| V$PAX_Q6 | 5 | 13 | 0.38 |
| V$PAX4_04 | 3 | 8 | 0.38 |
| V$AP1_Q6 | 1 | 3 | 0.33 |
| V$AP1_Q6_01 | 1 | 3 | 0.33 |
| V$AP1FJ_Q2 | 1 | 3 | 0.33 |
| V$CDP_02 | 1 | 3 | 0.33 |
| V$CEBP_Q2 | 2 | 6 | 0.33 |
| V$CREB_02 | 2 | 6 | 0.33 |
| V$CREB_Q3 | 2 | 6 | 0.33 |
| V$EGR_Q6 | 1 | 3 | 0.33 |
| V$FXR_Q3 | 1 | 3 | 0.33 |
| V$GATA3_02 | 1 | 3 | 0.33 |
| V$GATA3_03 | 1 | 3 | 0.33 |
| V$HMGIY_Q6 | 1 | 3 | 0.33 |
| V$MRF2_01 | 1 | 3 | 0.33 |
| V$MYB_Q3 | 1 | 3 | 0.33 |
| V$MYB_Q5_01 | 1 | 3 | 0.33 |
| V$MYB_Q6 | 1 | 3 | 0.33 |
| V$OCT1_01 | 1 | 3 | 0.33 |
| V$OSF2_Q6 | 1 | 3 | 0.33 |
| V$PAX3_B | 1 | 3 | 0.33 |
| V$HMGIY_Q3 | 3 | 10 | 0.30 |
| V$FOXD3_01 | 2 | 7 | 0.29 |
| V$GATA1_01 | 2 | 7 | 0.29 |
| V$AP1_01 | 2 | 8 | 0.25 |
| V$CDPCR3_01 | 2 | 8 | 0.25 |
| V$GATA2_01 | 1 | 4 | 0.25 |
| V$HFH3_01 | 1 | 4 | 0.25 |
| V$NKX25_01 | 1 | 4 | 0.25 |
| V$OCT1_Q6 | 1 | 4 | 0.25 |
| V$SOX_Q6 | 1 | 4 | 0.25 |
| V$TAL1BETAE47_01 | 1 | 4 | 0.25 |
| V$TAXCREB_01 | 1 | 4 | 0.25 |
| V$YY1_01 | 1 | 4 | 0.25 |
| V$ATF4_Q2 | 1 | 5 | 0.20 |
| V$CEBP_Q3 | 1 | 5 | 0.20 |
| V$FOX_Q2 | 1 | 5 | 0.20 |
| V$CETS1P54_02 | 1 | 7 | 0.14 |
| V$MAF_Q6_01 | 1 | 7 | 0.14 |
